# Ultraviolet radiation exposure to the face in patients with xeroderma pigmentosum and healthy controls: applying a novel methodology to define photoprotection behaviour*

**DOI:** 10.1111/bjd.20899

**Published:** 2022-02-24

**Authors:** R.P.E. Sarkany, M. Canfield, M. Morgan, L. Foster, K. Johnstone, K. Sainsbury, V. Araujo‐Soares, H.C. Wulf, J. Weinman, J. Walburn, S. Norton

**Affiliations:** ^1^ Xeroderma Pigmentosum Unit Guys and St Thomas’ NHS Foundation Trust London UK; ^2^ Health Psychology Section Institute of Psychiatry Psychology & Neuroscience King’s College London London UK; ^3^ School of Cancer and Pharmaceutical Sciences King’s College London London UK; ^4^ Population Health Institute Faculty of Medical Sciences Newcastle University Newcastle UK; ^5^ Health Technology and Services Research Technical Medical Centre University of Twente the Netherlands; ^6^ Department of Dermatology Bispebjerg University Hospital Copenhagen Denmark

## Abstract

**Background:**

In xeroderma pigmentosum (XP), the main means of preventing skin and eye cancers is extreme protection against ultraviolet radiation (UVR). Protection is most important for the face.

**Objectives:**

We aimed to assess how well patients with XP adhere to medical advice to protect against UVR by objectively estimating the mean daily dose of UVR to the face.

**Methods:**

We objectively estimated the UVR dose to the face in 36 patients with XP and 25 healthy individuals over 3 weeks in the summer. We used a new methodology which combined UVR dose measurements from a wrist‐worn dosimeter with an activity diary record of face photoprotection behaviour for each 15‐min period spent outside. A protection factor was associated with each behaviour, and the data were analysed using a negative binomial mixed‐effects model.

**Results:**

The mean daily UVR dose (weighted for DNA damage capacity) to the face in the patients with XP was 0·13 standard erythemal doses (SEDs) (mean in healthy individuals = 0·51 SED). There was wide variation between patients (range < 0·01–0·48 SED/day). Self‐caring adult patients had a very similar UVR dose to the face as cared‐for patients (0·13 vs. 0·12 SED/day), despite photoprotecting much more poorly when outside, because the self‐caring adults were outside in daylight much less.

**Conclusions:**

Photoprotection behaviour varies widely within the XP group indicating that nonadherence to photoprotection advice is a significant issue. The timing and duration of going outside are as important as photoprotective measures taken when outside, to determine the UVR exposure to the face. This new methodology will be of value in identifying the sources of UVR exposure in other conditions in which facial UVR exposure is a key outcome, particularly in patients with multiple nonmelanoma skin cancers.

## Abstract 2

**Linked Comment:** J.J. DiGiovanna and K.H. Kraemer. *Br J Dermatol* 2022; **186**:607–608.

Plain language summary available online

## Graphical abstract

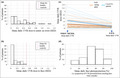

Xeroderma pigmentosum (XP) is a recessive disease characterized by skin cancers, ocular cancers and neurological degeneration.^1^ The mean life expectancy is 32 years of age in the USA^2^ and lower in tropical countries where invasive facial skin cancers cause death in adolescence. Eighty per cent of cases are due to defective nucleotide excision repair, and 20% result from defective translesion synthesis past ultraviolet‐induced DNA damage.^3^ There are eight causative genes, each corresponding to an XP complementation group (A–G and V). Clinical heterogeneity occurs between and within complementation groups.^1^

The main means of preventing eye and skin cancers is photoprotection. As 5% of daylight comprises ultraviolet radiation (UVR), this necessitates rigorous avoidance of and protection from UVR in daylight using protective visors, clothing, hats, gloves and sunscreens.^4^ Eighty per cent of skin cancers in XP are on the face, head and neck,^5^ and so face protection is critical.

Prior to our mixed‐methods programme of research,^6^ there had been no studies of how well patients with this disease photoprotect. Our recent studies in patients with XP, using subjective measures of UVR protection (questionnaire, interview and N‐of‐1 methodologies), have suggested that poor photoprotection may be widespread.^7^, ^8^, ^9^

In this study we assessed how well patients with XP photoprotect, by objectively estimating the UVR reaching their faces. Because the face is the key site clinically, the dose of UVR reaching the face is the relevant indicator of adherence to photoprotection.

There is no methodology to accurately and objectively estimate facial UVR exposure. Previous studies have either relied on self‐report measures of protection or have measured environmental UVR without accounting for protection. Therefore, we developed a new technique to objectively estimate UVR reaching the face by combining an existing technology to objectively measure UVR exposure at the wrist, with a self‐reported measure of face photoprotection. Here we use this new methodology to objectively estimate the daily dose of UVR to the face in a group of patients with XP for 3 weeks in the summer. We compare this with a group of healthy adults as a ‘benchmark’ for the new technique to see how the behaviour of the least‐adherent patients compares with this healthy group.

## Materials and methods

This study was part of a mixed‐methods programme of research.^6^ Approval to conduct it was granted by Camden and King’s Cross Research Ethics Committee 15/LO/1395.

### Participants

Patients were recruited from the UK National XP Clinic. XP was diagnosed from: reduced unscheduled DNA repair in cultured skin fibroblasts,^10^, ^11^ typical clinical features^1^ and genomic mutation analysis in all eight XP‐related genes.^1^

Eligible patients were contacted by a research nurse. For patients under 16 years of age, and adults lacking the capacity to consent (due to XP‐related cognitive impairment), their carer was contacted. Patients, or carers, were recruited only if they understood sufficient English to complete the questionnaires and diaries. Clinical heterogeneity and age range within the studied group reflect the heterogeneity of the disorder.

We also recruited 25 healthy adults (18 years of age and older) from King’s College London University staff and students via a volunteer recruitment email (without offering inducement). This number was chosen to be similar to the number of self‐caring adult patients with XP studied.

### Procedure

Each participant combined wearing a UVR electronic dosimeter (SunSaver 3^12^) on the wrist, with completing a ‘UVR protection diary’ to record face photoprotection activities for each 15‐min period spent outdoors. They did this throughout a 3‐week period during the months when environmental UVR levels are highest in the UK (6 May–6 August). Patients were supplied with their usual Sun Protection Factor (SPF) 50 high UVA protection sunscreen and had been trained to apply it by an XP clinic nurse. The 25 healthy controls followed the same procedure but used their own sunscreen, which could be of any SPF, without prior training.

Environmental UVR was recorded by solar monitoring stations nearest to each participant’s address (data provided by Public Health England).

### Measurements

#### Ultraviolet radiation dose to the wrist

The SunSaver 3 dosimeter measures UVR exposure^13^ at the wrist.^14^, ^15^, ^16^ Because its spectral response matches the erythema action spectrum,^17^ measurements are expressed as ‘standard erythemal doses’ (SEDs), a measure weighted to the erythema action spectrum.^18^ The erythema and DNA damage action spectra are similar,^19^ so dosimeter readings reflect the DNA damage potential of measured UVR, the clinically relevant measure in XP. A ‘data logger’, controlling the sensor, records UVR level, movement and temperature every 5 s and calculates mean UVR every 5 min. Participants were asked to wear the wrist dosimeter from 6am to 10pm, and to ensure that the watch was uncovered by slightly rolling up a long sleeve or wearing the watch over it.

#### Photoprotection activities

All participants completed a paper ‘UVR protection diary’ to record each 15‐min interval spent outside between 6am and 10pm during the 21 days (File S1; see Supporting Information). For non‐self‐caring adults and children in the XP group, the diary was completed by the main carer. The diary was based on the Office of National Statistics Time‐Use Survey,^20^ improving reliability and validity by including the duration of each behaviour. Each page represented 1 day, with daylight hours split into 15‐min segments. Participants indicated periods over 10 min spent outside (rounded to the nearest 15 min), recording face photoprotection behaviours used during that time (including wearing: visor, hat, hoodie ‘worn up’, glasses, scarf, face buff, sunscreen, lip‐block) and the activity they were doing.

### Analysis of data

#### Calculation of ultraviolet radiation dose to the face

As it is not possible to wear a dosimeter on the face, we calculated the dose to the face [‘UVR dose (face)’] by combining the recorded UVR dose at the wrist [‘UVR dose (wrist)’] with the record of face photoprotection behaviour in the diary for each 15‐min interval (three 5‐min dosimeter measurements were combined for mean UVR exposure over each 15 min, to match the 15‐min diary records).

For each 15‐min period, the UVR dose (face) was calculated by multiplying the UVR dose (wrist) by a ‘face protection factor’ (FPF) associated with the behaviour recorded in the diary. The FPF is the proportion of UVR prevented from reaching facial skin by that behaviour.

### Estimation of ‘face protection factors’

To account for behaviours selectively protecting different areas, the face was divided into five regions: forehead, nose and cheeks, chin and jaw, eyelids, lips; the first three contributed 30% each to the FPF and the last two 5% each. For each behaviour, we estimated the photoprotection for each region of the face and estimated the behaviour’s overall FPF by adding up the protection of each region corrected for its size (5% or 30%). These photoprotection factors for the whole face are listed in Table 1. No UVR penetrates the UVR protective full‐face visor (our unpublished data). Because the XP‐specific visor used by all the patients covers the whole face and the front of the neck the XP visor has an FPF of 1·0.

**Table 1:** ‘Face protection factors’ (FPF) corresponding to each photoprotective behaviour listed in the activity diary

Face photoprotective behaviour	FPF for conversion of UVR dose (wrist) to UVR dose (face) (1·0 = 100% protection; 0·0 = no protection)
UVR protective visor	1·0
No protection	0·0
Sunscreen application	0·0–0·79: value calculated from the time since sunscreen was last applied (0·79 immediately after application, decreasing in a linear manner to 0·0 after 8 h)
Hat	0·30
Glasses	0·05
Scarf or face buff	0·65
Hoodie worn up	0·30

UVR, ultraviolet radiation

Protection by a hat was modelled with a sample of hats provided by the patients. On average, in summer, at UK latitudes, the patients’ hats shaded the whole forehead (100% protection) but not the rest of the face (0% protection) leading to our estimated hat FPF of 0·3 (forehead estimated as 30% of facial skin by area).

Protection by a ‘hoodie worn up’ was modelled by observing that patients’ hoodies when ‘worn up’ covered the jaw and chin region (30% of facial skin by area) but not the rest of the face.

Protection by sunscreen/lip block was modelled with a reducing function over the time since the last application. UVB contributes much more erythemally effective energy than UVA in sunshine,^21^ so we modelled the sunscreen’s FPF for UVB and extrapolated to UVA. Sunscreen is typically applied at 0·4–1·0 mg cm^–2^.^22^ We have assumed that patients with XP also apply sunscreen at 0·8 mg cm^–2^. Because there is a logarithmic association between application thickness and SPF,^23^ SPF 50 sunscreen applied at 0·8 mg cm^–2^ provides an initial FPF of 0·79. (Even in the most closely supervised healthy subjects, application has never been observed to be thicker than 1·0 mg cm^–2^. If patients with XP were applying sunscreen more thickly than we have assumed, the logarithmic mathematics would reduce the impact of this error on the UVR dose to the face.) We modelled the decrease in sunscreen FPF over the time since last application on the basis of studies of persistence of applied sunscreen.^24^, ^25^ We assumed a linear decrease in protection over time, with no significant photoprotection by 8 h; hence, the estimated FPF for sunscreen of 0·79 immediately after application, decreasing linearly to 0·00 at 8 h after application.

### Demographic and clinical data

This information has been gathered from the medical records and questionnaire responses of these patients with XP.^8^ The questionnaire (File S2; see Supporting Information) was extensive, providing some of the demographic and clinical data presented here; our paper analysing the psychological and social data has been submitted for publication and is currently under review. Healthy subjects completed a short demographic and skin‐typing questionnaire.

### Statistical analysis of data

All analyses used a negative binomial mixed‐effects model, with the outcome being dosimetry records for each individual on each study day [i.e. UVR dose (wrist), calculated UVR dose (face), and time spent outside]. A random intercept was included to account for repeated observations across multiple days within individuals. All models controlled for weekend effects and total environmental (i.e. solar monitoring station) UVR measurements. Average daily total UVR exposure to the watch, UVR exposure of the face, and time outside were calculated for each patient using empirical Bayes estimates.

## Results

Of the 93 patients with XP known to the XP Service, 78 were eligible, 47 of whom consented to take part. Of these, six withdrew before dosimeter fitting, one provided < 14 days of data, in two patients their dosimeters malfunctioned, and two did not provide a full analysable dataset. Thirty‐six of the 47 patients recruited completed the whole study. Because of the rarity of the disease, the sample size was based on the maximum number of patients that could be recruited, rather than on a calculation of statistical power.

### Demographic and clinical characteristics

Table 2 shows the demographic and clinical characteristics of the 36 patients with XP. The age range was wide [mean age 29·2 years, range 5–63 years (SD 18·8)]. There were more males than females. Most patients (21 of 36) were self‐caring adults. Of the 15 non‐self‐caring patients, 11 were children, and four were cognitively impaired adults. All XP complementation groups were represented apart from B (which is very rare). XP‐related cognitive impairment and eye problems were common. Almost one‐half described abnormally severe sunburn reactions, in line with the mix of complementation groups (only groups A, B, D, F and G suffer severe sunburn reactions).^26^ Around two‐thirds of the patients had started photoprotection by age 13 years (mean 12·6, SD 13·2). Nearly 40% had suffered a mucocutaneous malignancy, occurring at widely differing ages, reflecting the clinical heterogeneity of XP.^1^

**Table 2:** Demographic and clinical characteristics of the patients with XP

	Adult self‐caring (*n* = 21)	Patients cared for by a caregiver^a^ (*n* = 15)	Total (*n* = 36)
*Demographic variables*			
Male, *n* (%)	14 (67%)	9 (60%)	23 (64%)
Female, *n* (%)	7 (33%)	6 (40%)	13 (36%)
Age (years), mean (SD)	40·0 (16·0)	14·1 (9·9)	29·2 (18·8)
*Clinical variables*			
Complementation group			
A	5 (24%)	3 (20%)	8 (22%)
C	6 (29%)	5 (33%)	11 (31%)
D	1 (5%)	4 (27%)	5 (14%)
E	2 (10%)	0 (0%)	2 (6%)
F	1 (5%)	0 (0%)	1 (3%)
G	0 (0%)	2 (13%)	2 (6%)
V	6 (28·6%)	0 (0%)	6 (17%)
Unknown	0 (0%)	1 (7%)	1 (3%)
Age at diagnosis (years), mean (SD)	20·4 (15·9)	4·2 (3·4)	13·7 (14·7)
Age at which patient started photoprotection (years), mean (SD)	18·9 (14·0)	3·7 (3·2)	12·6 (13·2)
Abnormal sunburn reaction (XP SSS 0–3^b^) *n* (%)			
SSS: 0	12 (63·1%)	5 (41·7%)	17 (54·8%)
SSS: 1	2 (10·5%)	0 (0%)	2 (6·5%)
SSS: 2	2 (10·5%)	2 (16·7%)	4 (12·9%)
SSS: 3	3 (15·8%)	5 (41·7%)	8 (25·8%)
Previous skin, eye or oral malignancy, *n* (%)	12 (57%)	2 (13%)	14 (39%)
Cognitive impairment, *n* (%)	2 (10%)	5 (33%)	7 (19%)
Visual problems, *n* (%)	13 (62%)	10 (66%)	23 (64%)

^a^Children and nonself‐caring cognitively impaired adults. ^b^XP SSS: Information available from only 31 of the patients. One point is given for each of: sunburn requiring medical consultation and/or treatment, sunburn occurring outside the months of March to September in the UK, sunburn taking longer than 72 h to resolve.^26^

SSS, sunburn severity score

The healthy subjects were 25 university staff (44% male, 56% female) with a mean age of 29·2 years (SD 6·34). There was a range of Fitzpatrick skin types: type II in five, type III in seven, type IV in nine, and type V in four.

### Dosimetry and activity diary data

The 36 patients provided a total of 775 days (mean 21·5 days) of data for which the dosimeter was worn and diary completed. Patients reported going outside on 665 of the 775 days (85·8%). The proportion of days when patients went outside ranged from 9·5% to 100% across the sample. On average patients spent 141 min outside/day, with a mean of 55 min outside daily during the period of highest UVR (11am–3pm).

The 25 healthy participants provided a total of 491 days of data (mean 19·6 days) for which the dosimeter was worn and diary completed. Intraindividual variation across days (SD 131·3) was larger than interindividual variation in this group (SD 89·9).

**Figure 1:**
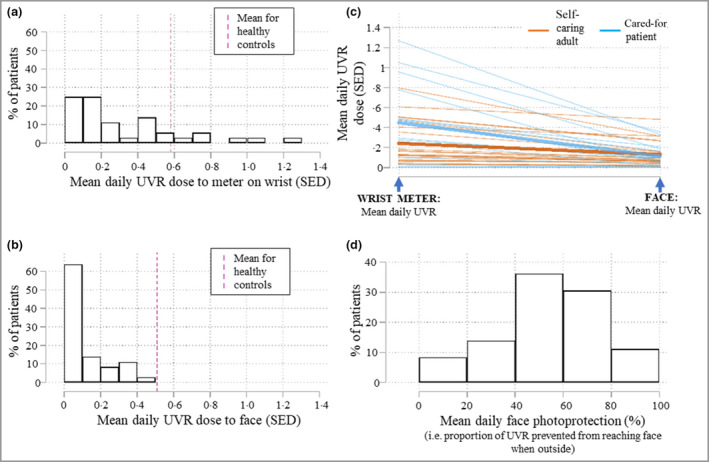
In the patients with xeroderma pigmentosum: (a) distribution of mean daily UVR to wrist dosimeter; (b) distribution of mean calculated daily UVR to the face; (c) individual differences between wrist dosimeter and face UVR exposure; (d) distribution of mean daily face photoprotection. SED, standard erythemal dose; UVR, ultraviolet radiation.

UVR exposure dosimetry data for the 36 patients with XP are displayed in Figure 1. The mean daily UVR (wrist) was 0·33 (median 0·22) SED, with a very wide range (< 0·01–1·27 SED) (Figure 1a). Variability in daily UVR exposure (wrist) within patients over time was smaller than between patients (SD_within_ 0·34; SD_between_ 0·63), with a within‐person repeatability intraclass correlation (r_ICC_) of 0·36, implying consistency of behaviour for individual patients over time. The mean daily total UVR (wrist) in the patients with XP (0·33 SED) was lower than in the healthy sample [0·58 SED; *t(59)* = –2·98, *P* = 0·004], although there were individual patients with XP with exposure higher than the healthy sample mean (Figure 1a). The mean daily environmental UVR from solar monitoring stations during the study period was 21·5 SED (SD 7·6; range 4·1–40·3 SED), so the mean daily UVR (wrist) exposure of the patients with XP represents 1·5% of the total environmental UVR.

In the healthy sample, there was large variation in behaviour across the group [SD of the UVR dose (wrist) 1·24, compared with a mean of 0·58]. The mean daily dose (wrist) on weekdays in the healthy group was much lower (0·41 SED, SD 0·05) than at weekends (0·99 SED, SD 0·14), with intraindividual variation in dose across days (SD 1·11) larger than interindividual variation (SD 0·55). Most healthy participants, 15 of 25 (60%), had a mean daily UVR dose (wrist) > 1·5 SED on at least 1 day.

Figure 1b shows the dose of UVR reaching the face in the patients with XP. The mean daily UVR dose (face) was 0·13 SED (median 0·08 SED) in the patients with XP, compared with 0·51 SED in the healthy sample [*t*(59) = –12·24, *P* < 0·001)]. Wide variation in UVR dose to the face in the XP group (< 0·01–0·48 SED) reflected variation across the group in both photoprotection and UVR dose at the wrist. Figure 1d shows the mean face photoprotection (i.e. percentage of UVR prevented from reaching the face) in the patients with XP. The variation in the degree to which patients with XP protected the face when outside ranged from < 10% to 95% (mean 52·4%).

### Comparison of self‐caring and nonself‐caring patients

Comparing the 21 self‐caring adult patients with XP with the 15 looked after by a carer (Table 3), the mean daily UVR exposure (wrist) was higher (0·45 vs. 0·24 SED) for those looked after by a carer (difference not quite reaching statistical significance [*t*(34) = –1·99, *P* = 0·055]). Figure 1c shows individual results in the XP group, differentiating self‐caring adults from patients looked after by a carer. Each line represents one patient and links their mean daily UVR dose (wrist) with the calculated mean daily UVR dose (face). The range of face UVR exposure was wide in both groups: 0·015–0·48 SED/day in self‐caring adults and 0·004–0·358 SED/day in cared‐for patients. The lines on the graph for the cared‐for sample are generally steeper because they were outside more, but had better photoprotection when outside, reducing mean UVR at the wrist (0·45 SED/day) to a mean dose reaching the face of 0·12 SED/day. Self‐caring adults were outside less [mean daily UVR (wrist) 0·24 SED/day] but protected less well when outside. The mean UVR dose (face) was almost identical for the self‐caring and cared‐for groups (0·13 vs. 0·12 SED/day). The large standard deviations within these subgroups result from the wide variation in behaviours within the XP group as a whole. These results fit with the finding in the whole XP group that mean daily photoprotection was generally higher in those with higher mean daily wrist UVR exposure (*r* = 0·73, *P* < 0·001), i.e. those who were outside more tended to photoprotect better.

**Table 3:** Ultraviolet radiation (UVR) photoprotection in self‐caring adult patients with xeroderma pigmentosum (XP) vs. cared‐for adults or children with XP

	Mean % (SD) of total UVR exposure protected against (from activity diaries)	Mean (SD) daily UVR dose measured at wrist (SED)	Mean (SD) daily calculated UVR dose to the face (SED)
Self‐caring adult patients with XP	43 (17)	0·24 (0·22)	0·13 (0·13)
Cared‐for adults or children with XP	66 (23)	0·45 (0·40)	0·12 (0·11)

SED, standard erythemal dose

## Discussion

We have developed a new technique to objectively estimate UVR exposure to the face. We have used it to explore levels of photoprotection in 36 patients with XP in mid‐summer. The UVR dose to the face is the clinically relevant outcome in XP, because most of the skin cancers are on the face.^5^ We also studied a group of 25 healthy adults as a comparative benchmark for the methodology and for the XP group. We identified wide variation in UVR dose to the face between patients with XP, showing a broad range in adherence to photoprotection advice. The patient with the highest mean daily UVR dose to the face had 120‐fold higher exposure than the patient with the lowest. The worst‐protecting patients had a dose to the face similar to the mean in the healthy group. These large differences between patients with XP in photoprotection behaviour are likely to be clinically significant.

The group of healthy adults is not a control group, and may not be entirely typical of the wider healthy population, but was a test of our methodology and a benchmark to see how the behaviour of the least‐adherent patients compared with these healthy individuals. In this London‐based healthy group, the UVR exposure was comparatively low (similar to findings in Copenhagen^27^), higher at weekends than on weekdays, and higher than in the patients with XP.

UVR dose to the face results from two variables: how much individuals go outside, and how well they protect when outside. Although there is wide variation between patients, our data suggest that, for self‐caring adult patients with XP, lower UVR doses to the face are achieved by spending more time inside rather than by photoprotecting well when outside, whereas children and cared‐for adult patients achieve a similar UVR dose to the face by photoprotecting better rather than by avoiding being outside. The implication is that behaviour change interventions and medical advice in XP need to focus on reducing UVR exposure by altering timing and duration of time spent outside, as well as the traditional focus on improving photoprotective measures taken when outside,^4^ and that they require tailoring for different patient subgroups.

Despite the high number of recorded observations (dosimeter and diary data recorded every 15 min for 21 days) and the recruitment of nearly half the known cases of XP in the UK, caution is needed when comparing UVR exposure between individuals and between subgroups within our patient group, as the sample was statistically small, particularly with the statistical implications of the wide variation in UVR behaviour within the group. A potential source of error is that the dosimeter, worn on the wrist, might underestimate exposure by missing times when covered by a sleeve. However, we trained participants to avoid this by rolling up the sleeve slightly or putting the watch over the sleeve. Overall, we consider that diary data combined with objective measurement of UVR exposure provide robust data for a clinically relevant outcome never previously explored in XP.

The dramatic variation in UVR exposure to the face between patients with XP has therapeutic implications. Nonadherence to photoprotection advice is likely to have a profound impact on prognosis in XP. We have previously identified factors underlying nonadherence to photoprotection (as indicated from questionnaire responses) in patients with XP.^8^ We have carried out a detailed analysis in this group of patients with XP of the factors associated with higher UVR dose to the face, and this is reported in the submitted paper mentioned above. As in other diseases, psychosocial factors (e.g. perceived necessity and concerns about photoprotection, the extent to which photoprotection is habitual, the level of confidence to protect) predict poor photoprotection. Because these factors are potentially reversible and can be modified by behaviour change interventions,^28^ we have designed an intervention and are testing it in a clinical trial in these patients.^29^

This new methodology for objectively estimating facial UVR exposure may be useful in other groups requiring facial photoprotection. Photoprotection behaviour is resistant to change^22^, ^30^ and this technique may be useful to study nonadherence to photoprotection in patients at high risk of non‐melanoma skin cancers. These cancers are common^31^; UVR is estimated to cause 80–83% of them^32^ and 71·6% occur on the face.

In conclusion, we have developed a technique to objectively estimate UVR exposure to the face and have used it in a group of patients with XP and in a group of healthy individuals. We identified wide variation in face photoprotection between patients with XP. These findings have therapeutic implications in XP. This technique may be useful in more common diseases in which reducing UVR exposure to the face is important.

## Author Contribution

**Robert Sarkany:** Conceptualization (lead); Funding acquisition (lead); Investigation (equal); Methodology (equal); Project administration (lead); Supervision (lead); Writing – original draft (lead); Writing – review & editing (lead). **Martha Canfield:** Data curation (supporting); Formal analysis (supporting); Writing – review & editing (supporting). **Myfanwy Morgan:** Formal analysis (supporting); Investigation (supporting); Methodology (supporting); Validation (supporting); Writing – review & editing (supporting). **Lesley Foster:** Data curation (supporting); Investigation (supporting); Writing – review & editing (supporting). **Katherine Johnstone:** Data curation (supporting); Investigation (supporting); Writing – review & editing (supporting). **Kirby Sainsbury:** Investigation (supporting); Methodology (supporting); Writing – review & editing (supporting). **Vera Araujo‐Soares:** Investigation (supporting); Methodology (supporting); Writing – review & editing (supporting). **Hans Christian Wulf:** Conceptualization (supporting); Funding acquisition (supporting); Investigation (supporting); Methodology (supporting); Resources (supporting); Supervision (supporting); Writing – review & editing (supporting). **John Weinman:** Conceptualization (lead); Funding acquisition (lead); Investigation (supporting); Methodology (lead); Project administration (lead); Resources (lead); Supervision (lead); Validation (supporting); Writing – review & editing (supporting). **Jessica Walburn:** Conceptualization (equal); Data curation (equal); Investigation (equal); Methodology (equal); Project administration (equal); Resources (equal); Supervision (supporting); Writing – review & editing (supporting). **Sam Norton:** Conceptualization (supporting); Data curation (equal); Formal analysis (lead); Investigation (equal); Methodology (equal); Project administration (equal); Writing – review & editing (supporting).

## Supporting information


**File S1** The photoprotection activity diary.

**File S2** The cross‐sectional questionnaire.

**Video S1** Author video.

**Powerpoint S1** Journal Club Slide Set.
