# Supplementary material for: Ultraviolet radiation exposure to the face in patients with xeroderma pigmentosum and healthy controls: applying a novel methodology to define photoprotection behaviour
Source: Br J Dermatol. 2022 Feb 24;186(4):713–20. doi: 10.1111/bjd.20899 (PMC9306996; doi:10.1111/bjd.20899)

Sarkany et al. Ultraviolet exposure in xeroderma pigmentosum

**File S1: The photoprotection activity diary**


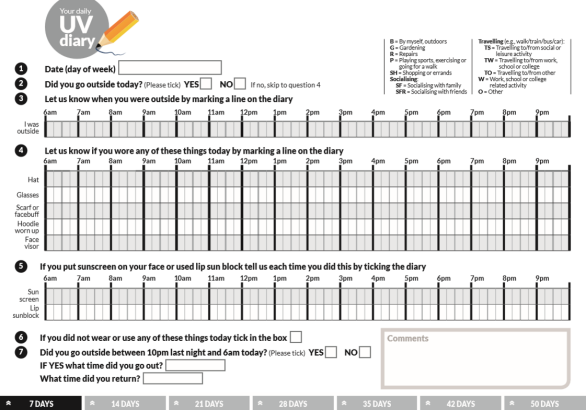

Supplement: Supplementary file 1 — File S1 The photoprotection activity diary. [file BJD-186-713-s003.docx]
