# Supplementary material for: Ultraviolet radiation exposure to the face in patients with xeroderma pigmentosum and healthy controls: applying a novel methodology to define photoprotection behaviour
Source: Br J Dermatol. 2022 Feb 24;186(4):713–20. doi: 10.1111/bjd.20899 (PMC9306996; doi:10.1111/bjd.20899)
Supplement: Supplementary file 2 — File S2 The cross‐sectional questionnaire. [file BJD-186-713-s004.docx]

| **Understanding Ultraviolet Protection (UV) in**  **Xeroderma Pigmentosum (XP) Questionnaire**  **Xeroderma Pigmentosum National Service**  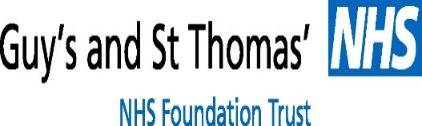**2^nd^ Floor, South Wing**  **St Thomas’ Hospital**  **Westminster Bridge Road**  **London SE1 7EH** |
| --- |

**We are trying to understand more about how having to protect against daylight affects the lives of people who have XP. From the answers we hope to be able to find ways to help patients and their families.**

**Please answer as many questions as you can. Where you are not sure of an answer, it is fine if you put your best guess.**

**Remember all the information you give will be treated with the strictest confidence and will be analysed anonymously. The people who look after you in the XP clinic will not see the answers you give.**

**If you are 12-15 years old and take responsibility for your own UV protection, please look through the questionnaire FIRST to see whether you feel able to complete it on your own. If you do not think you can, or if you are unsure, it is best for your parent or carer to complete one for you instead. They will complete a different version of questionnaire. Please contact our Research Nurse, Lesley Foster (Lesley.Foster@gstt.nhs.uk) if you do not have the version for parents or carers.**

**Thank you for taking the time to answer this questionnaire. Because XP is rare, every questionnaire that is completed and sent back to us makes a big difference for this research.**

**Filling out the questionnaire takes about 25 minutes. There are questions on both sides of each page.**

**The questionnaire is split into different topics:**

- **XP and your thoughts about it**
- **What you do to protect yourself from Ultraviolet (UV) in daylight**

Some questions ask about what you usually do and others ask about the last few days or weeks. Some questions look the same as others but are actually different, so please can you try to answer as accurately as you can.

- **Your views about UV protection**
- **General things about you and your quality of life**

**Before we ask about your XP, we’d like to find out a few details about you.**

1. How old are you now? _______________Years
2. Are you: (please tick) Male or Female
3. I am completing the questionnaire because: (please tick one option)

| I have been diagnosed with XP and I am 16 years or over |  |
| --- | --- |
| I have been diagnosed with XP and I am 12 - 15 years old and have chosen to complete the questionnaire myself |  |

1. Do you have any of these qualifications? (Please tick all that apply).

If you have no formal qualifications please tick the box below

| NVQ or equivalent |  |
| --- | --- |
| GCE / GCSE / O-level or equivalent |  |
| A-level or equivalent |  |
| Pre-degree foundation course/ diploma / HND or equivalent |  |
| Undergraduate degree |  |
| Masters |  |
| Doctorate/ PhD |  |
| Professional qualification |  |
| Other  Please write in________________________ |  |
| No Formal qualifications |  |

| **ABOUT YOUR XP** |
| --- |

**Please complete or circle the answer**

| 1. How old were you when you were diagnosed as having XP? | ……….…. Years |
| --- | --- |
| 1. Have you ever had a skin cancer? | Yes / No |
| 1. Have you had to see an Eye Specialist doctor because of any problems with the eyes? | Yes / No |
| 1. Has the XP caused problems with your hearing, walking, or speaking? | Yes / No |
| 1. Do you sunburn more easily than other people in your family who do not have XP? | Yes / No |
| 1. Have you ever had sunburn so badly you needed to see a doctor about it? | Yes / No |
| 1. Have you ever had sunburn in the winter in the country where you live? | Yes / No |
| 1. Have you ever had sunburn which took more than 3 days to go away? | Yes / No |
| 1. Do you take special measures to avoid some light bulbs? | Yes / No |

| 1. How would you describe your skin? |  |
| --- | --- |
|  | Fair or light coloured |
|  | Asian |
|  | Light brown |
|  | Afrocaribbean |
|  | Dark brown |
| 1. What colour are your eyes? |  |
|  | Blue |
|  | Green |
|  | Brown |
|  | Other |
| 1. Do you know what complementation group of XP you have? | Yes / No |
| 1. If **Yes** please circle the group |  |
|  | A |
|  | B |
|  | C |
|  | D |
|  | E |
|  | F |
|  | G |
|  | V |

**We are interested in your own personal views of how you see all aspects of your XP.**

**Please circle the number that best corresponds to your views:**

| 1. How much does your XP affect your life?   0 1 2 3 4 5 6 7 8 9 10  *no affect severely*  *at all affects my life* |
| --- |
| 1. How long do you think your XP will continue?   0 1 2 3 4 5 6 7 8 9 10  *a very forever*  *short time* |
| 1. How much control do you feel you have over the way XP affects your health?   0 1 2 3 4 5 6 7 8 9 10  *Absolutely extreme amount*  *no control of control* |
| 1. How much do you think daylight/UV protection can help your skin or eye health?   0 1 2 3 4 5 6 7 8 9 10  *not at all extremely helpful* |
| 1. How much do you think XP treatment in the clinic (e.g. surgery, creams) can help your skin or eye health?   0 1 2 3 4 5 6 7 8 9 10  *not at all extremely helpful* |
| 1. How much do you experience symptoms related to your XP?   0 1 2 3 4 5 6 7 8 9 10  *no symptoms many severe*  *at all symptoms* |
| 1. How concerned are you about your XP?   0 1 2 3 4 5 6 7 8 9 10  *not at all extremely*  *concerned concerned* |

**Please circle the number that best corresponds to your views:**

| 1. How well do you feel you understand your XP?   0 1 2 3 4 5 6 7 8 9 10  *don't understand understand*  *at all very clearly* |
| --- |
| 1. How much does your XP affect you emotionally? (does it make you angry, scared, upset or depressed?)   0 1 2 3 4 5 6 7 8 9 10  *not at all affected extremely affected*  *emotionally emotionally* |

**D THE YOU HAVE COMPLETED SECTION 1**

**YOU’RE ALMOST A THIRD OF THE WAY THROUGH YTHROUGH UO**

| **ABOUT YOUR PROTECTION FROM ULTRAVIOLET (UV) IN DAYLIGHT** |
| --- |

| 1. What is the date today? (day/month/year) | …………../……………/…………….. |
| --- | --- |

| 1. Which is your nearest town?   **If you do not wish to give this information please move to the next question.** |  |
| --- | --- |

**These questions focus on the LAST 7 DAYS**

| 1. Over the last 7 days, what is your best guess for **how many days** it has been sunny for at least half the day? | ………………………..Days (out of 7) |
| --- | --- |

| 1. Thinking about a **typical day** in the last 7 days, what is your best guess on average for the **number of hours** you have spent outside in daylight (with or without UV protection)? | ………………….hours per day |
| --- | --- |

| 1. We know it can be difficult to protect against UV all the time. Over the last 7 days when you went outside how often did you protect yourself against UV?   (Please circle the answer) | Never  rarely  sometimes  often  always |
| --- | --- |

| 1. Do you have UV protective window film on home windows? (Please circle the answer) | I have protective film on: ALL windows  SOME windows  NO windows  I don’t know |
| --- | --- |

| 1. Over the last 7 days, when at home to what extent have you stayed in rooms with UV protection   (Please circle the answer) | Not at all  rarely  sometimes  often  always |
| --- | --- |

| 1. Is there UV protective window film on windows at school or work?   (Please circle the answer) | There is film on:  ALL windows  SOME windows  NO windows  I don’t know  I don’t work or go to school outside the home |
| --- | --- |

| 1. Over the last 7 days, when at work or school to what extent have you stayed in rooms with UV protection   (Please circle the answer) | Not at all  rarely  sometimes  often  always |
| --- | --- |

| 1. Do you have UV protective window film on your car’s windows?   (Please circle the answer) | There is film on:  ALL windows  SOME windows  NO windows  I don’t know  I don’t have a car |
| --- | --- |

**We know it can be difficult to protect against UV in daylight all the time.**

**These items ask how much you agree or disagree with the statements about the different things you could do to protect yourself from UV.**

**They are very similar statements about each way you could have protected yourself over the PAST 7 DAYS. Please read each question carefully and circle the number that best corresponds to your views. If you did not protect from UV in that way please circle the box “I did not…”**

**OVER THE LAST 7 DAYS…..**

| 1. ***“Avoiding going outside*** *during the day was something I did automatically without thinking”*   1 2 3 4 5 6 7  Strongly Disagree Strongly Agree | I did not avoid going outside |
| --- | --- |
| 1. *“Every time I got ready to go outside,* ***wearing a face visor*** *was something I did automatically without thinking*”   1 2 3 4 5 6 7  Strongly Disagree Strongly Agree | I did not wear a face visor |
| 1. *“Every time I got ready to go outside,* ***wearing a hat*** *was something I did automatically without thinking”*   1 2 3 4 5 6 7  Strongly Disagree Strongly Agree | I did not wear a hat |
| 1. *“Every time I got ready to go outside* ***wearing glasses*** *was something I did automatically without thinking”*   1 2 3 4 5 6 7 Strongly Disagree Strongly Agree | I did not wear glasses |
| 1. *“Every time I got ready to go outside,* ***wearing sunscreen*** *was something I did automatically without thinking”*     1 2 3 4 5 6 7 Strongly Disagree Strongly Agree | I did not wear sunscreen |
| 1. *“Every time I got ready to go outside,* ***wearing lip sunblock*** *was something I did automatically without thinking”*   1 2 3 4 5 6 7 Strongly Disagree Strongly Agree | I did not wear lip sunblock |
| 1. *“Every time I got ready to go outside,* ***wearing a scarf or “face buff”*** *was something I did automatically without thinking”*   1 2 3 4 5 6 7  Strongly Disagree Strongly Agree | I did not wear a scarf or “face buff” |
| 1. *“Every time I got ready to go outside,* ***wearing a hoodie (worn up)*** *was something I did automatically without thinking”*   1 2 3 4 5 6 7  Strongly Disagree Strongly Agree | I did not wear a hoodie (worn-up) |
| 1. *“Every time I got ready to go outside,* ***wearing long sleeves**** *was something I did automatically without thinking”*   1 2 3 4 5 6 7  Strongly Disagree Strongly Agree | I did not wear long sleeves |
| 1. *“Every time I got ready to go outside,* ***wearing gloves*** *was something I did automatically without thinking”*   1 2 3 4 5 6 7  Strongly Disagree Strongly Agree | I did not wear gloves |
| 1. *“Every time I got ready to go outside,* ***wearing long trousers or thick tights*** *was something I did automatically without thinking”*     1 2 3 4 5 6 7  Strongly Disagree Strongly Agree | I did not wear long trousers or thick tights |

****sleeves would include coats or jackets***

**OVER THE LAST 7 DAYS WHEN OUTSIDE…..**

| 1. Thinking about all the things you did to protect yourself over the past 7 days (e.g., wearing sunscreen, wearing a hat), how well do you think they protected you from UV? (Please circle).   *Completely very well a fair amount a little Not*  *at all* | I did not do anything to protect myself from UV in the last 7 days |
| --- | --- |

**We know that people adapt their UV protection to fit in with everyday life.**

**These questions are about what you USUALLY do throughout the year to protect yourself from UV in daylight when it is CLOUDY AND then what you do on days when it is SUNNY.**

**Please circle how often you do the different things on the days when it is CLOUDY and then on the days when it is SUNNY, throughout the year.**

| 1. To what extent do you try to avoid going outside during the day? | **Cloudy days** | **Sunny days** |
| --- | --- | --- |
|  | Never  rarely  sometimes  often  always | Never  rarely  sometimes  often  always |

**WHEN YOU GO OUTSIDE… (Please circle the answer)**

|  | **Cloudy days** | **Sunny days** |
| --- | --- | --- |
| **Thinking about your FACE….** | | |
| 1. How often do you wear a face visor? | Never  rarely  sometimes  often  always | Never  rarely  sometimes  often  always |
| 1. How often do you wear a hat? | Never  rarely  sometimes  often  always | Never  rarely  sometimes  often  always |
| 1. How often do you wear glasses? | Never  rarely  sometimes  often  always | Never  rarely  sometimes  often  always |
| **When you go outside ……**  **(Please circle the answer)** | **Cloudy days** | **Sunny days** |
| 1. How often do you use sunscreen on your face? | Never  rarely  sometimes  often  always | Never  rarely  sometimes  often  always |
| 1. If you use sunscreen on your face, what sun protection factor does it have?   (or circle I do not….) | SPF number ......... | SPF number ......... |
|  | I do not wear sunscreen on my face | |
| 1. How often do you reapply the sunscreen on your face during the day?   (or circle I do not….) | ……………..x/day | ……………..x/day |
|  | I do not wear sunscreen on my face | |
| 1. How often do you put on lip sunblock? | Never  rarely  sometimes  often  always | Never  rarely  sometimes  often  always |
| 1. How often do you wear a scarf or “face buff”? | Never  rarely  sometimes  often  always | Never  rarely  sometimes  often  always |

| **When you go outside ……**  **(Please circle the answer)** | **Cloudy days** | **Sunny days** |
| --- | --- | --- |
| 1. How often do you wear a hoodie (worn -up)? | Never  rarely  sometimes  often  always | Never  rarely  sometimes  often  always |
| **Thinking about your ARMS OR HANDS…** | | |
| 1. How often do you wear long sleeves? | Never  rarely  sometimes  often  always | Never  rarely  sometimes  often  always |
| 1. How often do you use sunscreen on your arms or hands? | Never  rarely  sometimes  often  always | Never  rarely  sometimes  often  always |
| 1. How often do you wear gloves? | Never  rarely  sometimes  often  always | Never  rarely  sometimes  often  always |
| **Thinking about your LEGS…** | | |
| 1. How often do you wear long trousers or thick tights? | Never  rarely  sometimes  often  always | Never  rarely  sometimes  often  always |

| **When you go outside ……**  **(Please circle the answer)** | **Cloudy days** | **Sunny days** |
| --- | --- | --- |
| 1. How often do you use sunscreen on your legs? | Never  rarely  sometimes  often  always | Never  Rarely  sometimes  often  always |
| **Thinking about using sunscreen on your BODY (NOT FACE), ARMS, HANDS OR LEGS….** | | |
| 1. If you use sunscreen on any of these areas (body, arms, hands or legs) what sun protection factor does it have?   (or circle I do not….) | SPF number ......... | SPF number ......... |
|  | I do not wear sunscreen on my body, arms, hands or legs | |
| 1. How often do you reapply the sunscreen to any of these areas (body, arms, hands or legs) during the day?   (or circle I do not….) | ……………..x/day | ……………..x/day |
|  | I do not wear sunscreen on my body, arms, hands or legs | |

**HE**

**YOU’RE OVER HALF OF THE WAY THROUGH**

**NOT MUCH MORE TO GO..YTHROUGH UO**

**We would like to ask your personal views about protecting against UV in daylight. This means whatever you do to protect yourself, including:**

- **avoiding going outside during the day**
- **putting on sunscreen and lip sunblock**
- **wearing glasses**
- **wearing protective clothes (e.g., hat, scarf or face-buff, gloves, long sleeves, long trousers/thick tights)**
- **or wearing a face visor**

**These are statements other people have made about their UV protection.**

**Please indicate the extent to which you agree or disagree with what they have said by circling the answer that best corresponds to your views.**

**There are no right or wrong answers. We are interested in YOUR PERSONAL VIEWS.**

| 1. *“My health depends on protecting against UV”*   Strongly Agree Agree Uncertain Disagree Strongly Disagree |
| --- |
| 1. *“Having to protect against UV worries me”*   Strongly Agree Agree Uncertain Disagree Strongly Disagree |
| 1. *“My life would be impossible without protecting against UV”*   Strongly Agree Agree Uncertain Disagree Strongly Disagree |
| 1. *“If I did not protect against UV I would be very ill”*   Strongly Agree Agree Uncertain Disagree Strongly Disagree |
| 1. *“I sometimes worry about other people’s reactions to the things I have to do to protect* *against UV (e.g., wearing a visor, using sunscreen, wearing a hat)”*   Strongly Agree Agree Uncertain Disagree Strongly Disagree |
| 1. *“I sometimes worry about long-term effects of having to protect against UV”*   Strongly Agree Agree Uncertain Disagree Strongly Disagree |

| 1. *“I don’t really understand why I need to protect against UV”*   Strongly Agree Agree Uncertain Disagree Strongly Disagree |
| --- |
| 1. *“My health in the future will depend on protecting against UV”*   Strongly Agree Agree Uncertain Disagree Strongly Disagree |
| 1. *“Having to protect against UV disrupts my life”*   Strongly Agree Agree Uncertain Disagree Strongly Disagree |
| 1. *“Protecting against UV stops my health getting worse”*   Strongly Agree Agree Uncertain Disagree Strongly Disagree |
| 1. *“I sometimes worry about the impact on family and friends of having to protect against UV”*   Strongly Agree Agree Uncertain Disagree Strongly Disagree |

**We are now asking about the NEXT 7 DAYS.**

**These questions are about the different things you might do to protect yourself from UV. We know that people want to do some things more than others, and they find some things easier to do than others. We want to find out what YOU THINK.**

**These statements are very similar to each other about the ways you might protect yourself over the next 7 days. Please read each question carefully and think about each way in turn.**

| 1. Over the next 7 days I intend to protect myself by **avoiding going outside** during the daytime   1 2 3 4 5 6 7  Strongly Disagree Strongly Agree |
| --- |

**When I am outside in the NEXT 7 DAYS….**

**(If you are not intending to go outside in the next 7 days please skip to question 88.)**

| 1. I intend to protect myself by **wearing a face visor**     1 2 3 4 5 6 7  Strongly Disagree Strongly Agree |
| --- |
| 1. I intend to protect myself by **wearing a hat**     1 2 3 4 5 6 7  Strongly Disagree Strongly Agree |
| 1. I intend to protect myself by **wearing glasses**   1 2 3 4 5 6 7  Strongly Disagree Strongly Agree |
| 1. I intend to protect myself by **putting on sunscreen**   1 2 3 4 5 6 7  Strongly Disagree Strongly Agree |

**When I am outside in the NEXT 7 DAYS….**

| 1. I intend to protect myself by **putting on lip sunblock**   1 2 3 4 5 6 7  Strongly Disagree Strongly Agree |
| --- |
| 1. I intend to protect myself by **wearing a scarf or “face buff”**   1 2 3 4 5 6 7  Strongly Disagree Strongly Agree |
| 1. I intend to protect myself by **wearing a hoodie (worn-up)**   1 2 3 4 5 6 7  Strongly Disagree Strongly Agree |
| 1. I intend to protect myself by **wearing long sleeves**   1 2 3 4 5 6 7  Strongly Disagree Strongly Agree |
| 1. I intend to protect myself by **wearing gloves**   1 2 3 4 5 6 7  Strongly Disagree Strongly Agree |
| 1. I intend to protect myself by **wearing long trousers or thick tights**   1 2 3 4 5 6 7  Strongly Disagree Strongly Agree |

***"We know these questions are repetitive…thank you for keeping going"***

**Over the NEXT 7 DAYS…..**

| 1. I am confident I could **avoid going outside** during the daytime   1 2 3 4 5 6 7  Strongly Disagree Strongly Agree |
| --- |

**When I am outside in the NEXT 7 DAYS….**

**(If you are not intending to go outside in the next 7 days please skip to question 99.)**

| 1. I am confident I could **wear a face visor**   1 2 3 4 5 6 7  Strongly Disagree Strongly Agree |
| --- |
| 1. I am confident I could **wear a hat**   1 2 3 4 5 6 7  Strongly Disagree Strongly Agree |
| 1. I am confident I could **wear glasses**   1 2 3 4 5 6 7  Strongly Disagree Strongly Agree |

**When I am outside in the NEXT 7 DAYS….**

| 1. I am confident I could put on **sunscreen**   1 2 3 4 5 6 7  Strongly Disagree Strongly Agree |
| --- |
| 1. I am confident I could put on **lip sunblock**   1 2 3 4 5 6 7  Strongly Disagree Strongly Agree |
| 1. I am confident I could **wear a scarf or “face buff”**   1 2 3 4 5 6 7  Strongly Disagree Strongly Agree |
| 1. I am confident I could **wear a hoodie (worn-up)**   1 2 3 4 5 6 7  Strongly Disagree Strongly Agree |

| 1. I am confident I could **wear long sleeves**   1 2 3 4 5 6 7  Strongly Disagree Strongly Agree |
| --- |
| 1. I am confident I could **wear gloves**   1 2 3 4 5 6 7  Strongly Disagree Strongly Agree |
| 1. I am confident I could easily **wear long trousers or thick tights**   1 2 3 4 5 6 7  Strongly Disagree Strongly Agree |

**Overall in the NEXT 7 DAYS………**

| 1. I am confident I can do the things I want to do to protect myself from UV     1 2 3 4 5 6 7  Strongly Disagree Strongly Agree |
| --- |

**About your XP and the people around you……**

| 1. How much support or help do you have from the people around you with your UV protection?   *No support hardly any support Some support lots of support comprehensive support* |
| --- |

| 1. How satisfied are you with the support or help that you have to help you with your UV protection?   *Very dissatisfied fairly dissatisfied uncertain fairly satisfied very satisfied* |
| --- |

**HE YOU’RE NOW IN THE FINAL SECTION**

**YOU’VE ALMOST FINISHED !**

**UO**

| **ABOUT YOU AND YOUR QUALITY OF LIFE** |
| --- |

**These questions are about you, your thoughts about the present and future, your emotions and your quality of life.**

**Please circle the number that best corresponds to your views:**

| 1. *“Thinking about the future is pleasant to me”*   1 2 3 4 5  Very true of me Very untrue of me |
| --- |
| 1. *“When I want to achieve something, I set goals and consider specific means of reaching those goals”*   1 2 3 4 5  Very true of me Very untrue of me |
| 1. *“Meeting tomorrow’s deadlines and doing other necessary work comes before tonight’s play”*   1 2 3 4 5  Very true of me Very untrue of me |
| 1. *“It seems to me that my future plans are pretty well laid out”*   1 2 3 4 5  Very true of me Very untrue of me |
| 1. *“I think that it seems useless to plan too far ahead because things hardly ever come out the way you planned anyway”*   1 2 3 4 5  Very true of me Very untrue of me |
| 1. *“If I do not get done on time, I do not worry about it”*   1 2 3 4 5  Very true of me Very untrue of me |
| 1. *“I try to live one day at a time”*   1 2 3 4 5  Very true of me Very untrue of me |
| 1. *“I feel that it is more important to enjoy what you are doing than to get the work done on time”*   1 2 3 4 5  Very true of me Very untrue of me |
| 1. *“I do not do things that are good for me if they do not feel good now”*   1 2 3 4 5  Very true of me Very untrue of me |

**Below are some statements about feelings and thoughts.**

**Please circle the answer that best describes your experience of each over the LAST 2 WEEKS**

| 1. *“I’ve been feeling optimistic about the future”*   None of the time Rarely Some of the time Often All of the time |
| --- |
| 1. *“I’ve been feeling useful”*   None of the time Rarely Some of the time Often All of the time |
| 1. *“I’ve been feeling relaxed”*   None of the time Rarely Some of the time Often All of the time |
| 1. *“I’ve been dealing with problems well”*   None of the time Rarely Some of the time Often All of the time |
| 1. *“I’ve been thinking clearly”*   None of the time Rarely Some of the time Often All of the time |
| 1. *“I’ve been feeling close to other people”*   None of the time Rarely Some of the time Often All of the time |
| 1. *“I’ve been able to make up my own mind about things”*   None of the time Rarely Some of the time Often All of the time |

**These questions are about how your quality of life is affected by the XP.**

| **Under each heading, please tick the ONE box that best describes your health TODAY.** | |
| --- | --- |
| 1. MOBILITY |  |
| I have no problems in walking about | ❑ |
| I have slight problems in walking about | ❑ |
| I have moderate problems in walking about | ❑ |
| I have severe problems in walking about | ❑ |
| I am unable to walk about | ❑ |
| 1. SELF-CARE |  |
| I have no problems washing or dressing myself | ❑ |
| I have slight problems washing or dressing myself | ❑ |
| I have moderate problems washing or dressing myself | ❑ |
| I have severe problems washing or dressing myself | ❑ |
| I am unable to wash or dress myself | ❑ |
| 1. USUAL ACTIVITIES (e.g. work, study, housework, family or leisure activities) |  |
| I have no problems doing my usual activities | ❑ |
| I have slight problems doing my usual activities | ❑ |
| I have moderate problems doing my usual activities | ❑ |
| I have severe problems doing my usual activities | ❑ |
| I am unable to do my usual activities | ❑ |
| 1. PAIN / DISCOMFORT |  |
| I have no pain or discomfort | ❑ |
| I have slight pain or discomfort | ❑ |
| I have moderate pain or discomfort | ❑ |
| I have severe pain or discomfort | ❑ |
| I have extreme pain or discomfort | ❑ |

| 1. ANXIETY / DEPRESSION |  |
| --- | --- |
| I am not anxious or depressed | ❑ |
| I am slightly anxious or depressed | ❑ |
| I am moderately anxious or depressed | ❑ |
| I am severely anxious or depressed | ❑ |
| I am extremely anxious or depressed | ❑ |

**The best health you can imagine**

10

0

20

30

40

50

60

80

70

90

100

5

15

25

35

45

55

75

65

85

95

| We would like to know how good or bad your health is TODAY. |
| --- |
| This scale is numbered from 0 to 100. |
| 100 means the best health you can imagine. 0 means the worst health you can imagine. |
| Mark an X on the scale to indicate how your health is TODAY. |
| Now, please write the number you marked on the scale in the box below. |

1. **YOUR HEALTH TODAY** =

**The worst health you can imagine**

| **ONE MORE QUESTION ABOUT YOU…** |
| --- |

**This is an extra question, some people may not want to fill it out. If you don’t want to do this question, just leave it blank.**

1. Which of the following **best** describes your **ethnicity?** Please tick **one** of the following options.

| **White**  British |  | **Black or Black British**  Caribbean |  |
| --- | --- | --- | --- |
| Any other White background  please write in__________________ |  | African |  |
| **Mixed**  White and Black Caribbean  White and Black African  White and Asian  Any other Mixed background  please write in__________________ |  | Any other Black background,  please write in_________________  **Chinese or other ethnic group**  Chinese  Any other  Please write in__________________ |  |
| **Asian or Asian British** |  |  |  |
| Indian |  |  |  |
| Pakistani |  |  |  |
| Bangladeshi |  |  |  |
| Any other Asian background  please write in__________________ |  |  |  |

**If you have any additional thoughts or comments please can you write them here:**

|  |
| --- |

**THANK YOU VERY MUCH FOR COMPLETING OUR QUESTIONNAIRE.**

**If you have any questions about the questionnaire please contact our Research Nurse Lesley Foster.**

Nurse Lesley Foster, National Xeroderma Pigmentosum Service

Floor 2, Block C, South Wing

St. John’s Institute of Dermatology

St Thomas' Hospital

Westminster Bridge Road

London SE1 7EH

Nurse Lesley Foster

Mobile: 07775111823

E-mail: [Lesley.Foster@gstt.nhs.uk](mailto:Lesley.Foster@gstt.nhs.uk)

Fax: 020 7188 1621
