# Supplementary material for: Ultraviolet radiation exposure to the face in patients with xeroderma pigmentosum and healthy controls: applying a novel methodology to define photoprotection behaviour
Source: Br J Dermatol. 2022 Feb 24;186(4):713–20. doi: 10.1111/bjd.20899 (PMC9306996; doi:10.1111/bjd.20899)
Supplement: Supplementary file 4 — Powerpoint S1 Journal Club Slide Set. [file BJD-186-713-s001.pptx]

## Slide 1
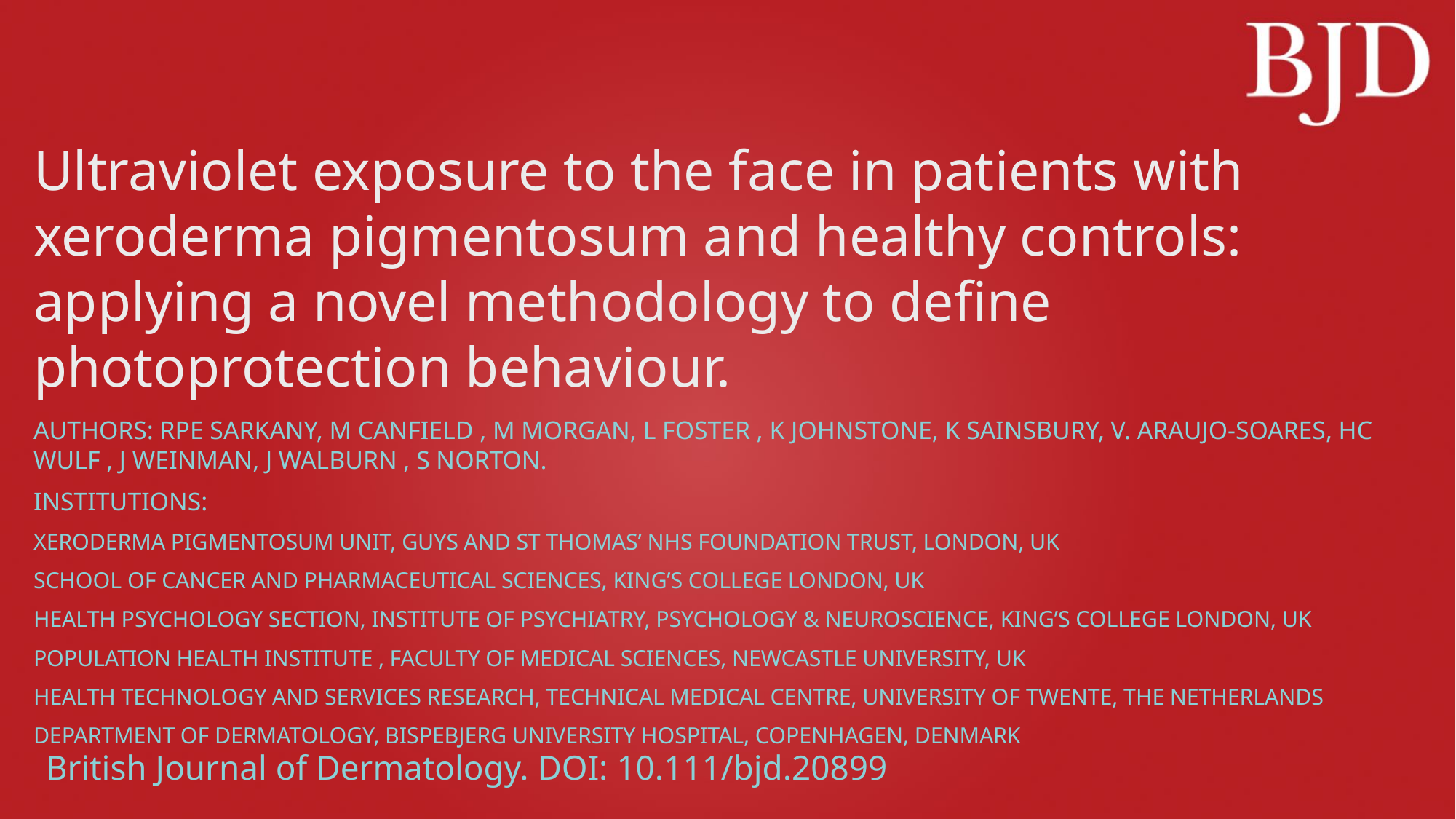

# Ultraviolet exposure to the face in patients with xeroderma pigmentosum and healthy controls: applying a novel methodology to define photoprotection behaviour.
AUTHORS: RPE Sarkany, M Canfield , M Morgan, L Foster , K Johnstone, K Sainsbury, V. Araujo-Soares, HC Wulf , J Weinman, J Walburn , S Norton.
INSTITUTIONS:
Xeroderma Pigmentosum Unit, Guys and St Thomas’ NHS Foundation Trust, london, uk
School of Cancer and Pharmaceutical Sciences, King’s College London, UK
Health Psychology Section, Institute of Psychiatry, Psychology & Neuroscience, King’s College London, UK
Population Health Institute , Faculty of Medical Sciences, Newcastle University, UK
Health Technology and Services Research, Technical Medical Centre, University of Twente, The Netherlands
Department of Dermatology, Bispebjerg University Hospital, Copenhagen, Denmark
British Journal of Dermatology. DOI: 10.111/bjd.20899

## Slide 2
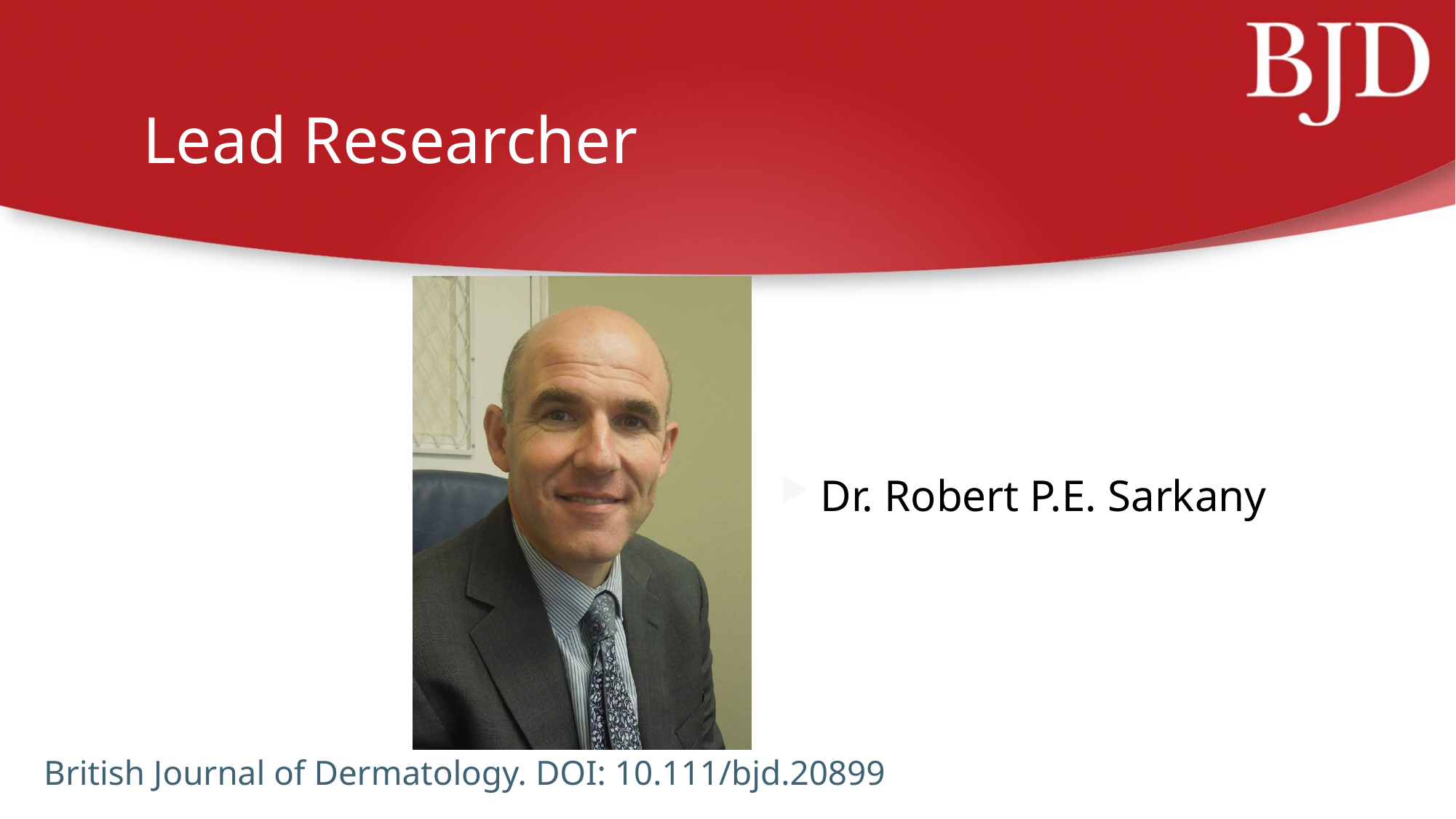

# Lead Researcher
Dr. Robert P.E. Sarkany
British Journal of Dermatology. DOI: 10.111/bjd.20899

## Slide 3
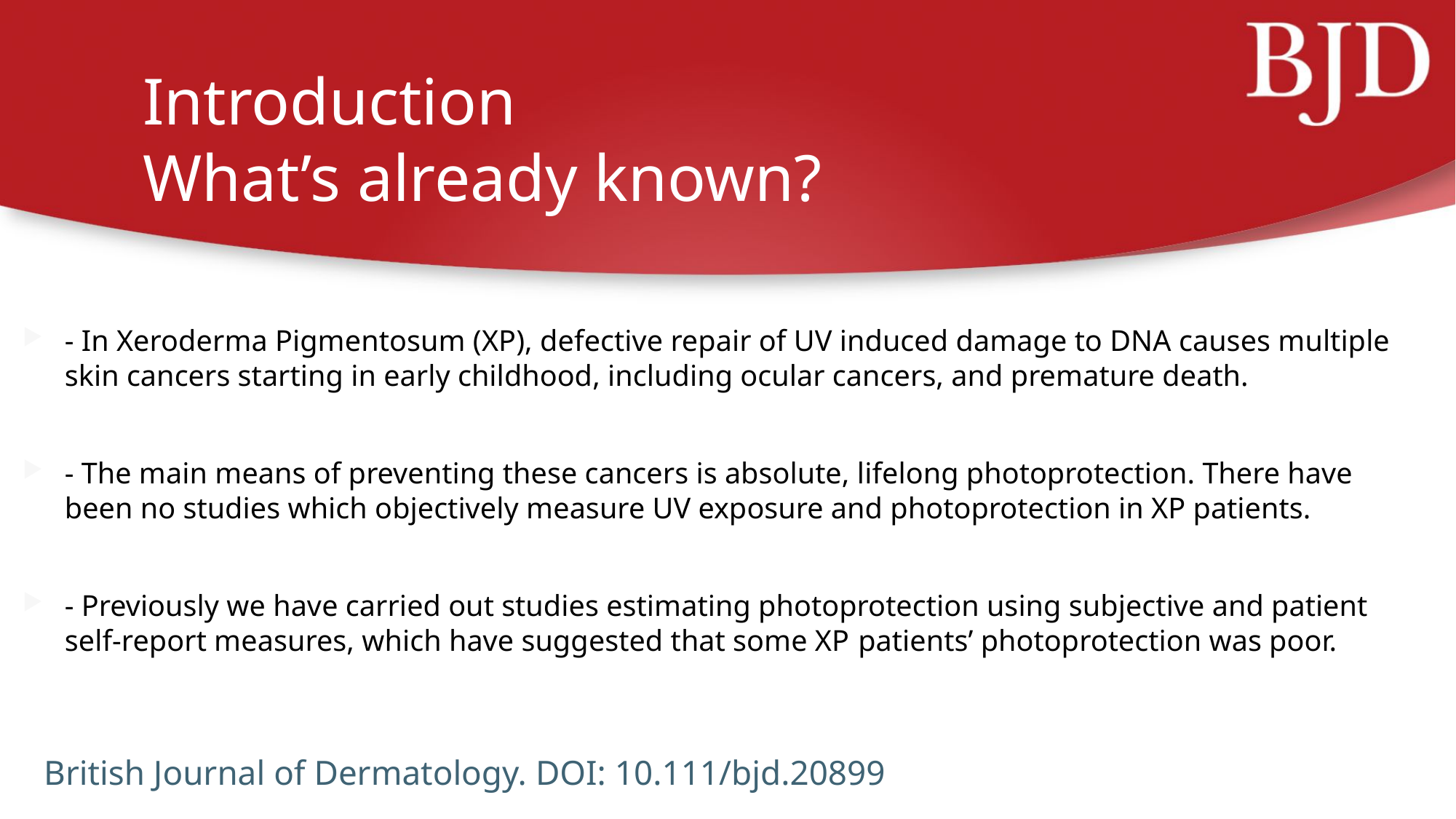

# Introduction What’s already known?
- In Xeroderma Pigmentosum (XP), defective repair of UV induced damage to DNA causes multiple skin cancers starting in early childhood, including ocular cancers, and premature death.
- The main means of preventing these cancers is absolute, lifelong photoprotection. There have been no studies which objectively measure UV exposure and photoprotection in XP patients.
- Previously we have carried out studies estimating photoprotection using subjective and patient self-report measures, which have suggested that some XP patients’ photoprotection was poor.
British Journal of Dermatology. DOI: 10.111/bjd.20899

## Slide 4
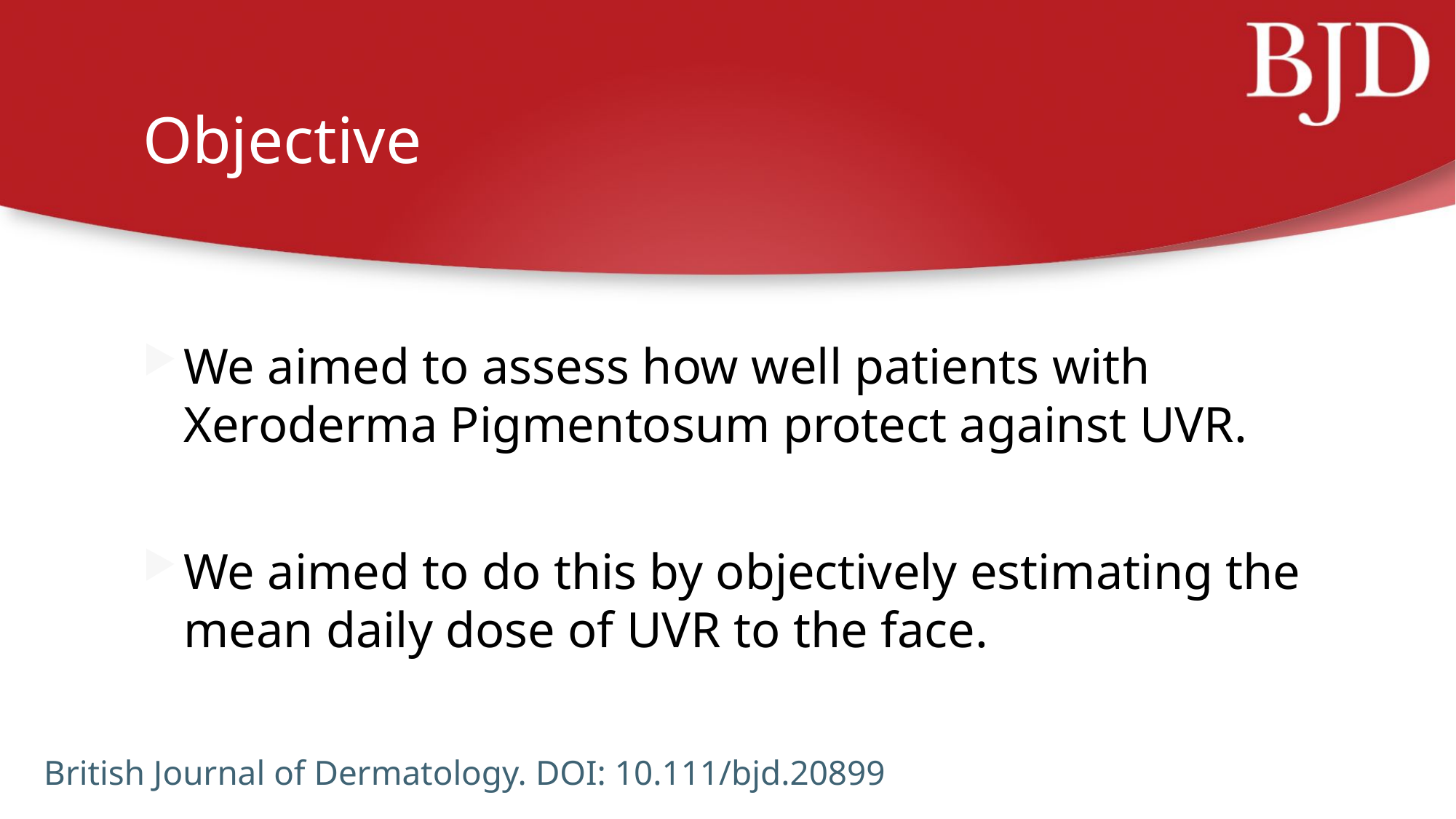

# Objective
We aimed to assess how well patients with Xeroderma Pigmentosum protect against UVR.
We aimed to do this by objectively estimating the mean daily dose of UVR to the face.
British Journal of Dermatology. DOI: 10.111/bjd.20899

## Slide 5
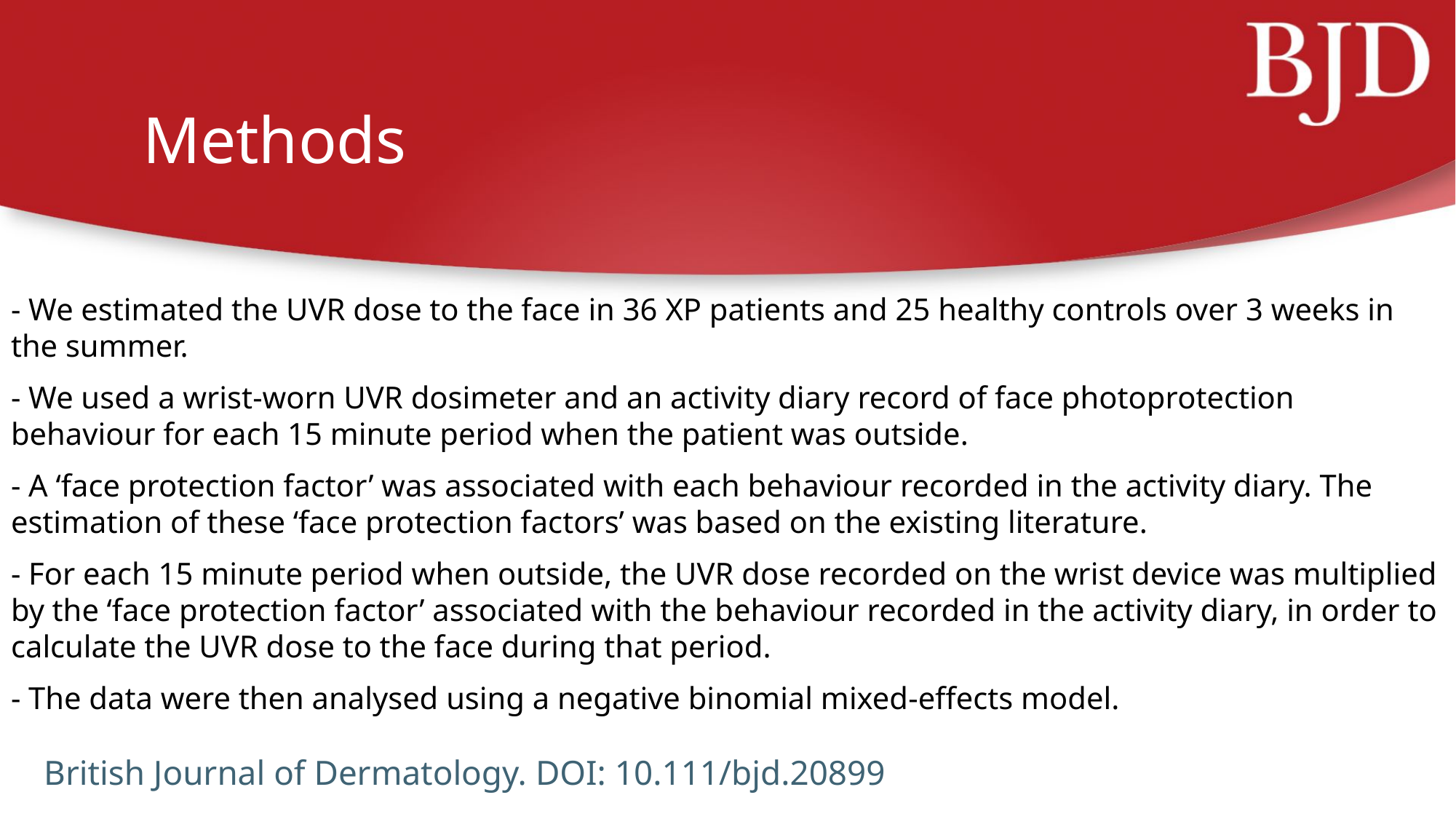

# Methods
- We estimated the UVR dose to the face in 36 XP patients and 25 healthy controls over 3 weeks in the summer.
- We used a wrist-worn UVR dosimeter and an activity diary record of face photoprotection behaviour for each 15 minute period when the patient was outside.
- A ‘face protection factor’ was associated with each behaviour recorded in the activity diary. The estimation of these ‘face protection factors’ was based on the existing literature.
- For each 15 minute period when outside, the UVR dose recorded on the wrist device was multiplied by the ‘face protection factor’ associated with the behaviour recorded in the activity diary, in order to calculate the UVR dose to the face during that period.
- The data were then analysed using a negative binomial mixed-effects model.
British Journal of Dermatology. DOI: 10.111/bjd.20899

## Slide 6
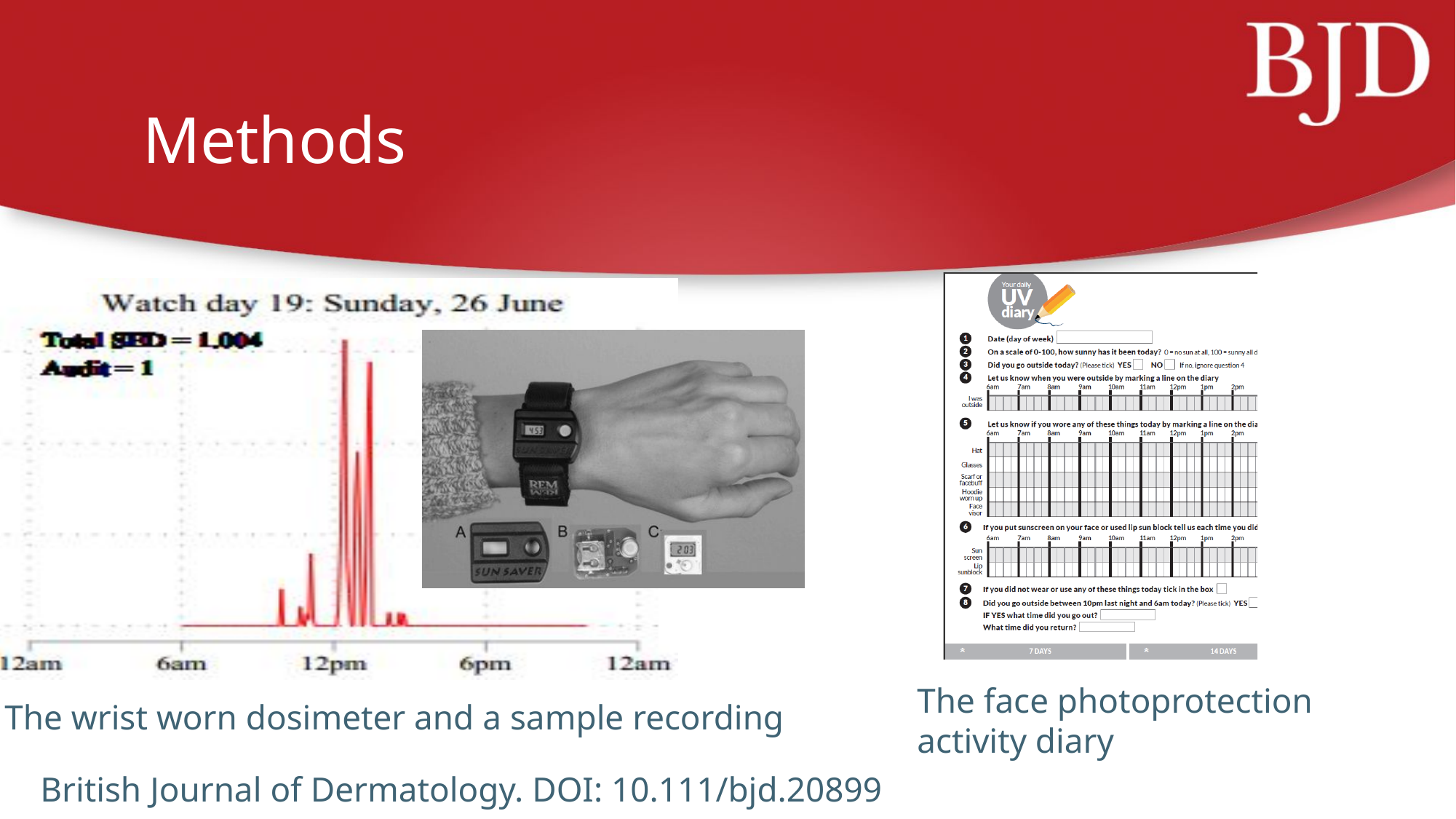

# Methods
Please include a maximum of 3 slides
The face photoprotection activity diary
The wrist worn dosimeter and a sample recording
British Journal of Dermatology. DOI: 10.111/bjd.20899

## Slide 7
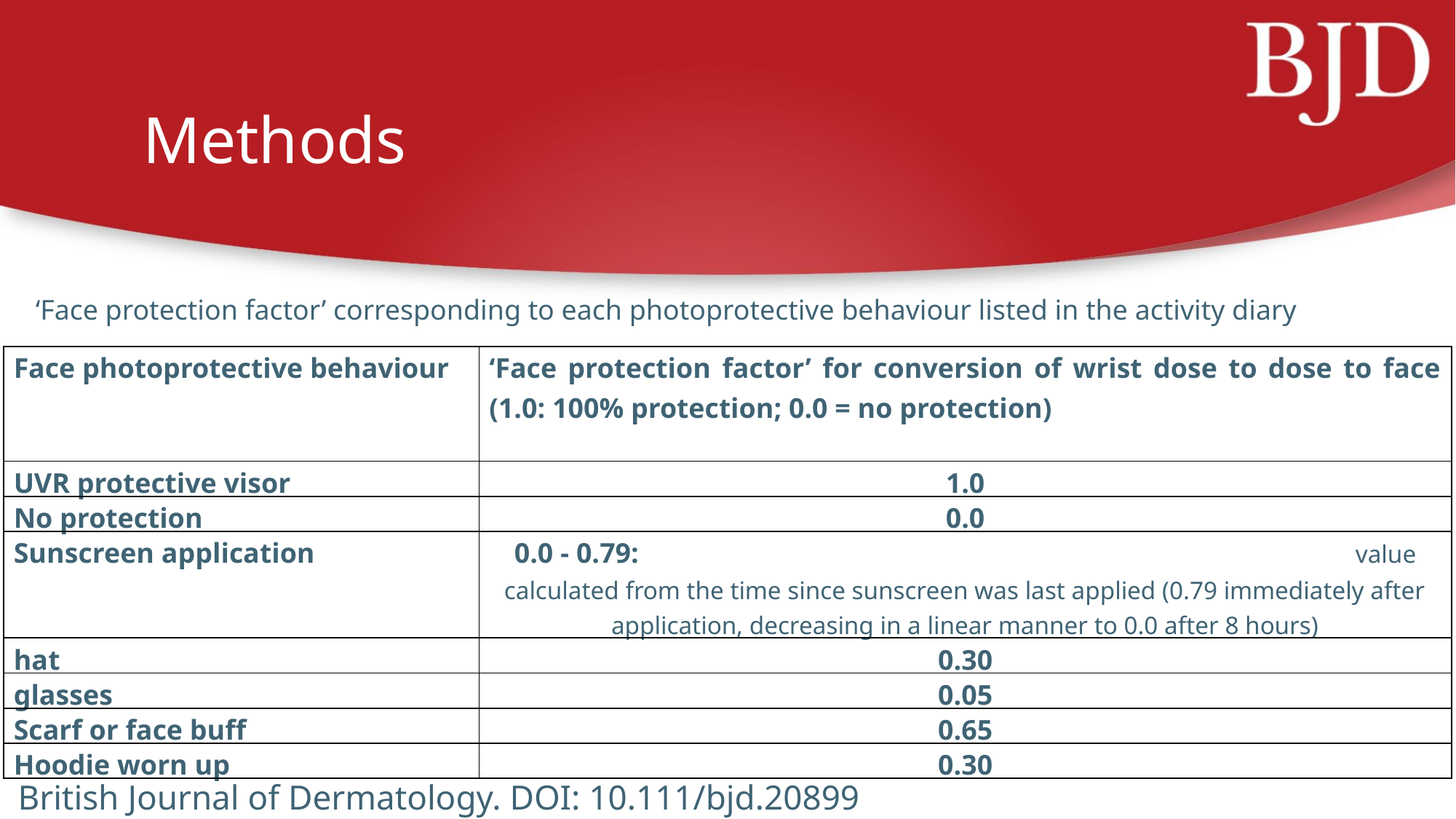

# Methods
‘Face protection factor’ corresponding to each photoprotective behaviour listed in the activity diary
| Face photoprotective behaviour | ‘Face protection factor’ for conversion of wrist dose to dose to face (1.0: 100% protection; 0.0 = no protection) |
| --- | --- |
| UVR protective visor | 1.0 |
| No protection | 0.0 |
| Sunscreen application | 0.0 - 0.79: value calculated from the time since sunscreen was last applied (0.79 immediately after application, decreasing in a linear manner to 0.0 after 8 hours) |
| hat | 0.30 |
| glasses | 0.05 |
| Scarf or face buff | 0.65 |
| Hoodie worn up | 0.30 |
British Journal of Dermatology. DOI: 10.111/bjd.20899

## Slide 8
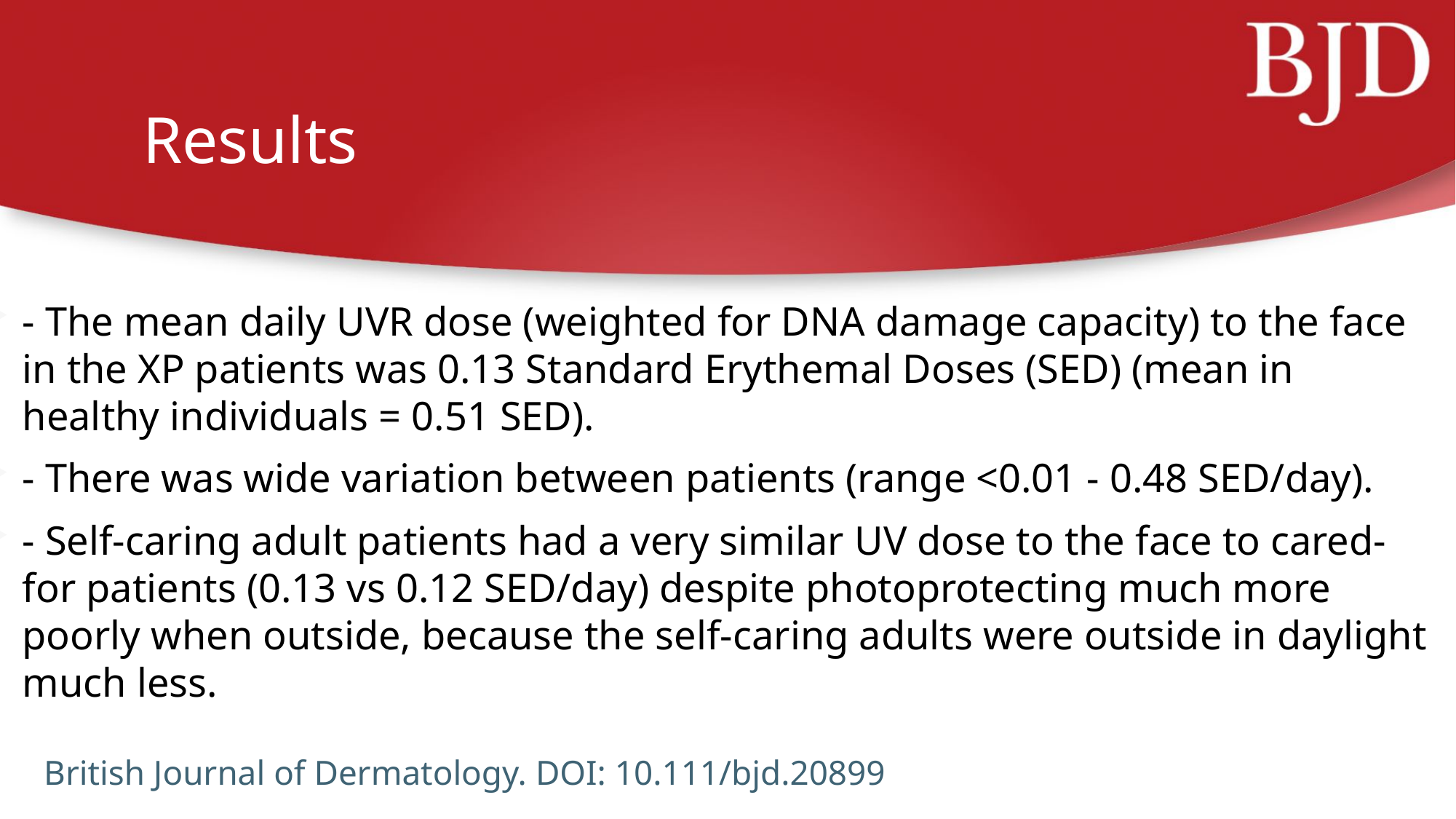

# Results
- The mean daily UVR dose (weighted for DNA damage capacity) to the face in the XP patients was 0.13 Standard Erythemal Doses (SED) (mean in healthy individuals = 0.51 SED).
- There was wide variation between patients (range <0.01 - 0.48 SED/day).
- Self-caring adult patients had a very similar UV dose to the face to cared-for patients (0.13 vs 0.12 SED/day) despite photoprotecting much more poorly when outside, because the self-caring adults were outside in daylight much less.
British Journal of Dermatology. DOI: 10.111/bjd.20899

## Slide 9
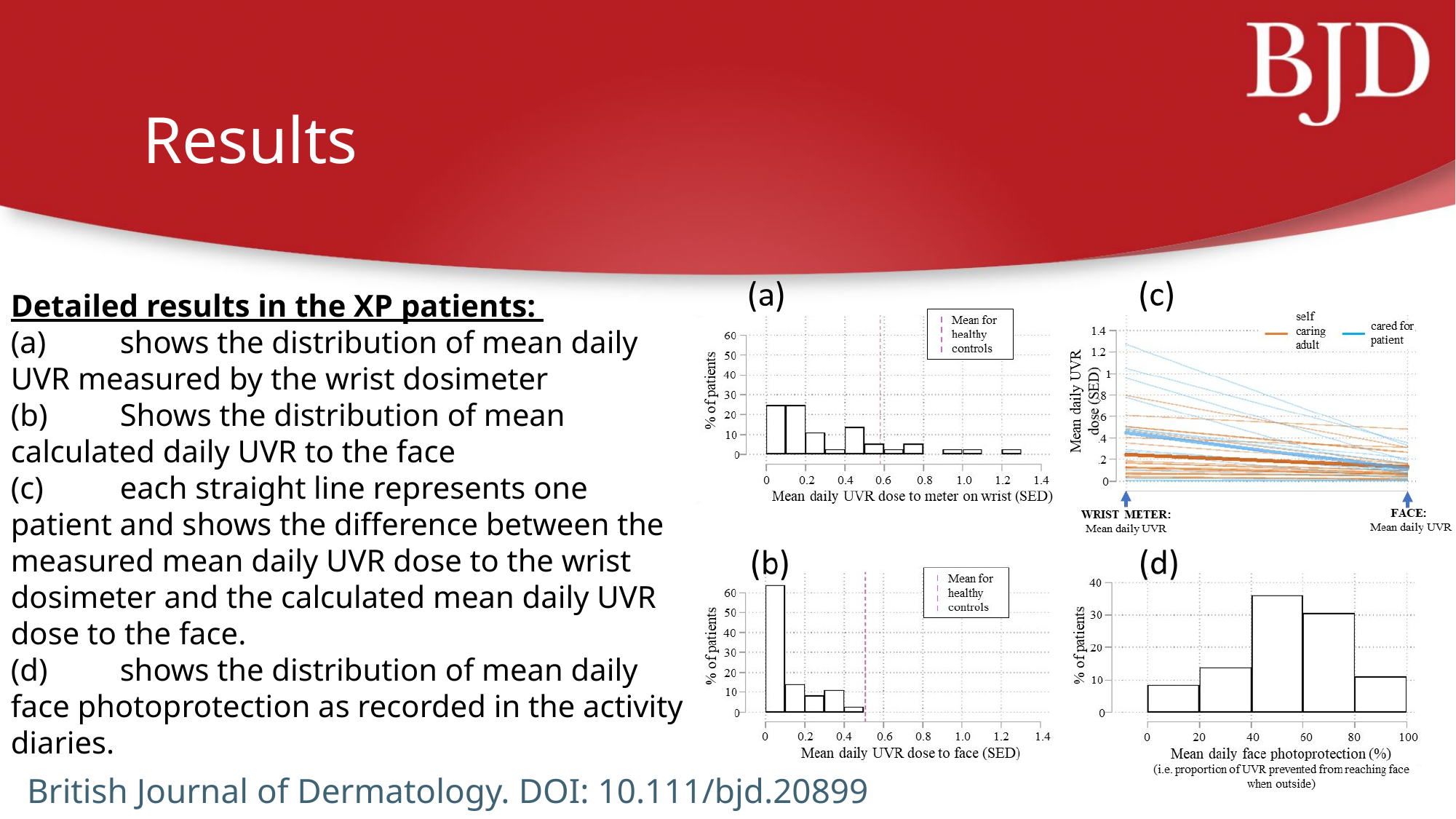

# Results
Detailed results in the XP patients:
(a)	shows the distribution of mean daily UVR measured by the wrist dosimeter
(b)	Shows the distribution of mean calculated daily UVR to the face
(c)	each straight line represents one patient and shows the difference between the measured mean daily UVR dose to the wrist dosimeter and the calculated mean daily UVR dose to the face.
(d)	shows the distribution of mean daily face photoprotection as recorded in the activity diaries.
British Journal of Dermatology. DOI: 10.111/bjd.20899

## Slide 10
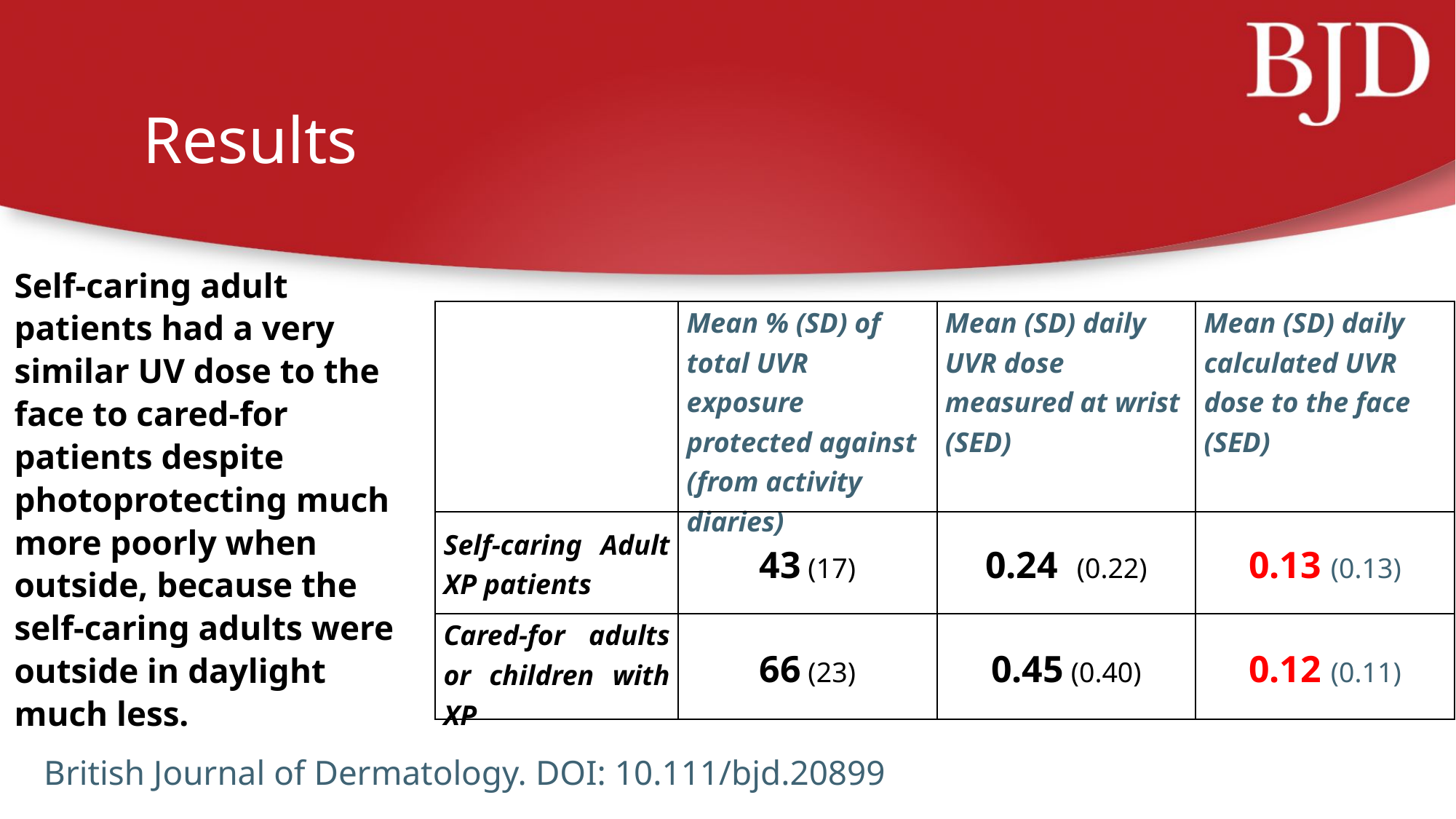

# Results
Self-caring adult patients had a very similar UV dose to the face to cared-for patients despite photoprotecting much more poorly when outside, because the self-caring adults were outside in daylight much less.
| | Mean % (SD) of total UVR exposure protected against (from activity diaries) | Mean (SD) daily UVR dose measured at wrist (SED) | Mean (SD) daily calculated UVR dose to the face (SED) |
| --- | --- | --- | --- |
| Self-caring Adult XP patients | 43 (17) | 0.24 (0.22) | 0.13 (0.13) |
| Cared-for adults or children with XP | 66 (23) | 0.45 (0.40) | 0.12 (0.11) |
British Journal of Dermatology. DOI: 10.111/bjd.20899

## Slide 11
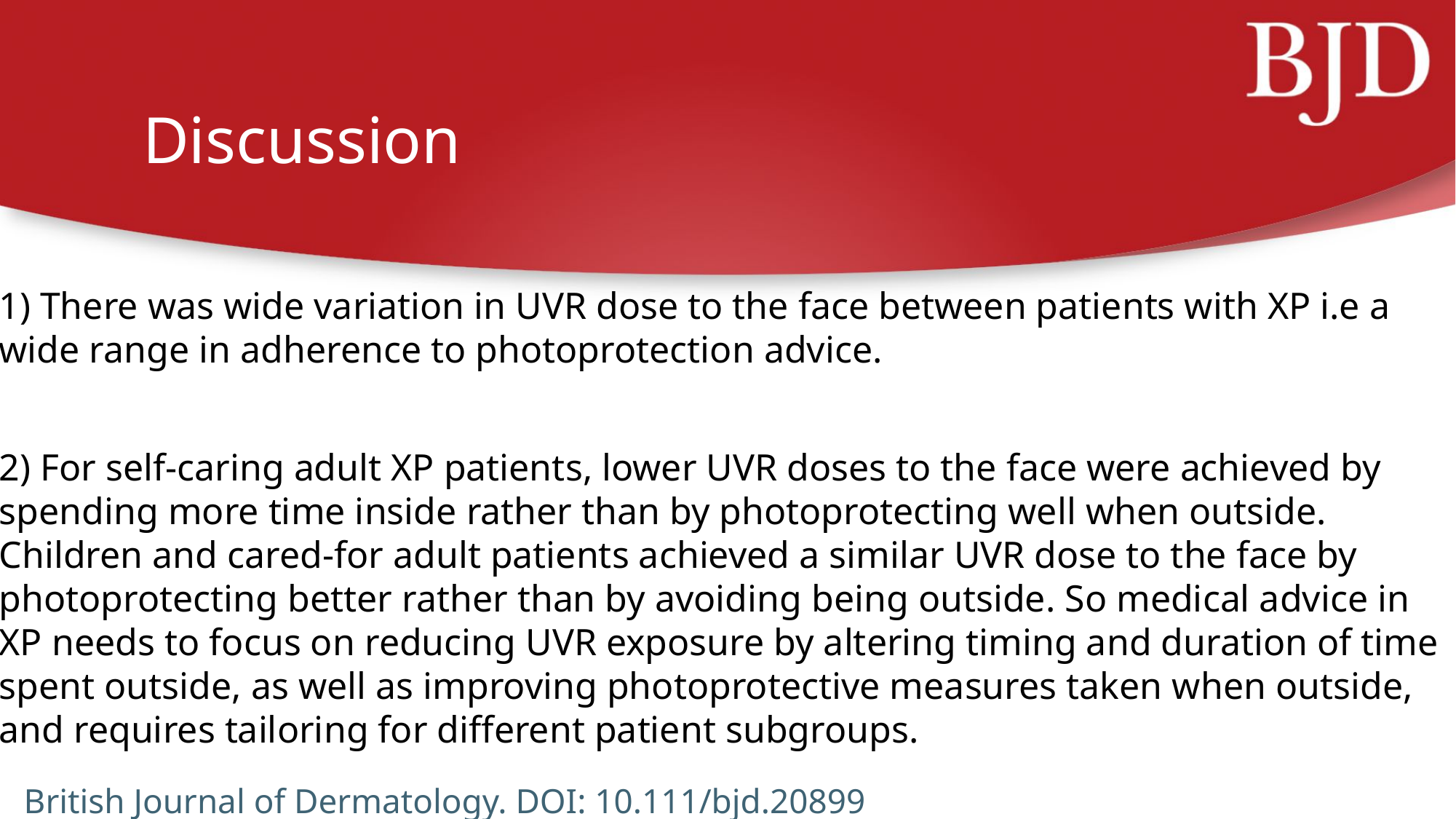

# Discussion
1) There was wide variation in UVR dose to the face between patients with XP i.e a wide range in adherence to photoprotection advice.
2) For self-caring adult XP patients, lower UVR doses to the face were achieved by spending more time inside rather than by photoprotecting well when outside. Children and cared-for adult patients achieved a similar UVR dose to the face by photoprotecting better rather than by avoiding being outside. So medical advice in XP needs to focus on reducing UVR exposure by altering timing and duration of time spent outside, as well as improving photoprotective measures taken when outside, and requires tailoring for different patient subgroups.
British Journal of Dermatology. DOI: 10.111/bjd.20899

## Slide 12
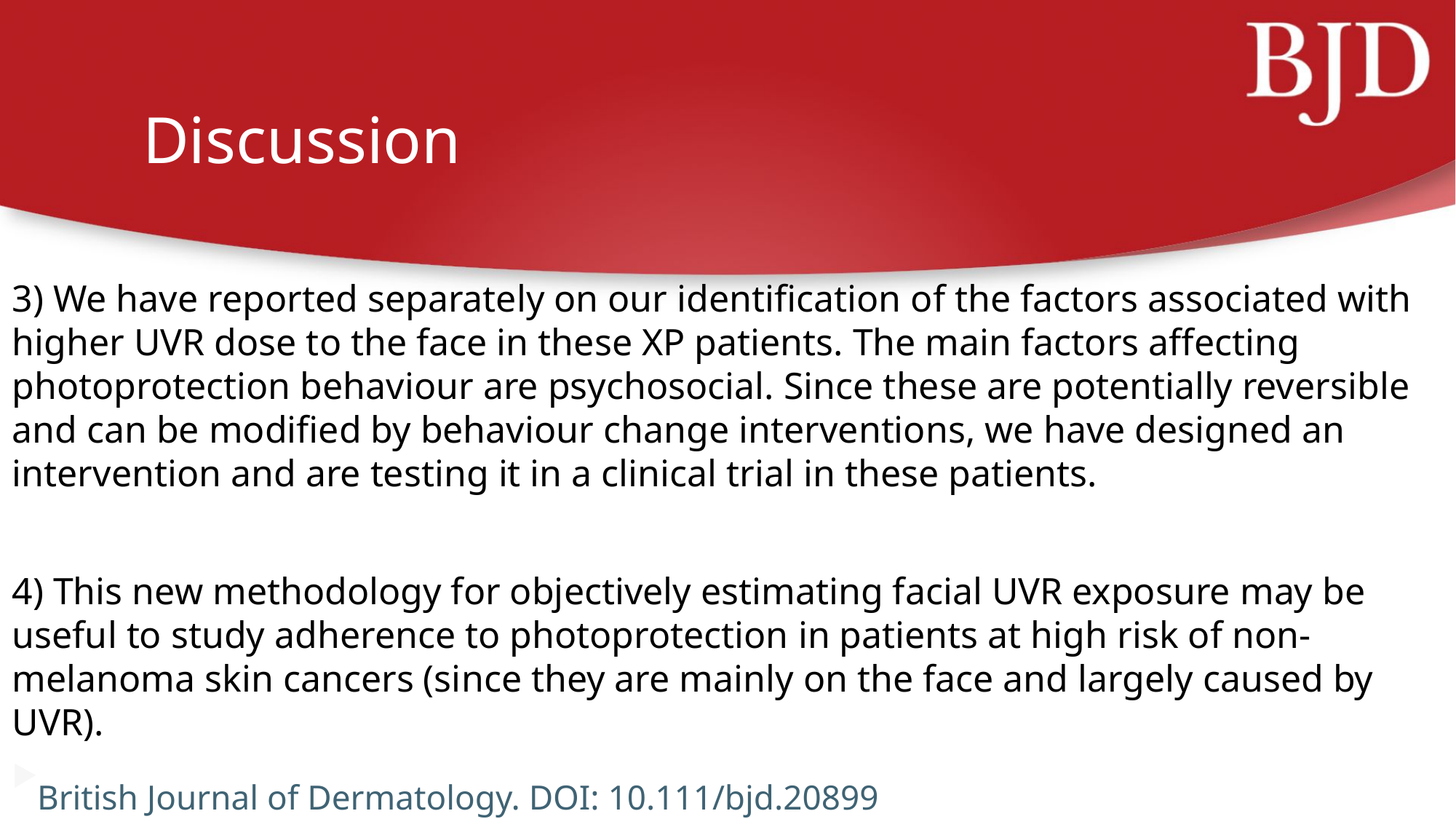

# Discussion
3) We have reported separately on our identification of the factors associated with higher UVR dose to the face in these XP patients. The main factors affecting photoprotection behaviour are psychosocial. Since these are potentially reversible and can be modified by behaviour change interventions, we have designed an intervention and are testing it in a clinical trial in these patients.
4) This new methodology for objectively estimating facial UVR exposure may be useful to study adherence to photoprotection in patients at high risk of non-melanoma skin cancers (since they are mainly on the face and largely caused by UVR).
British Journal of Dermatology. DOI: 10.111/bjd.20899

## Slide 13
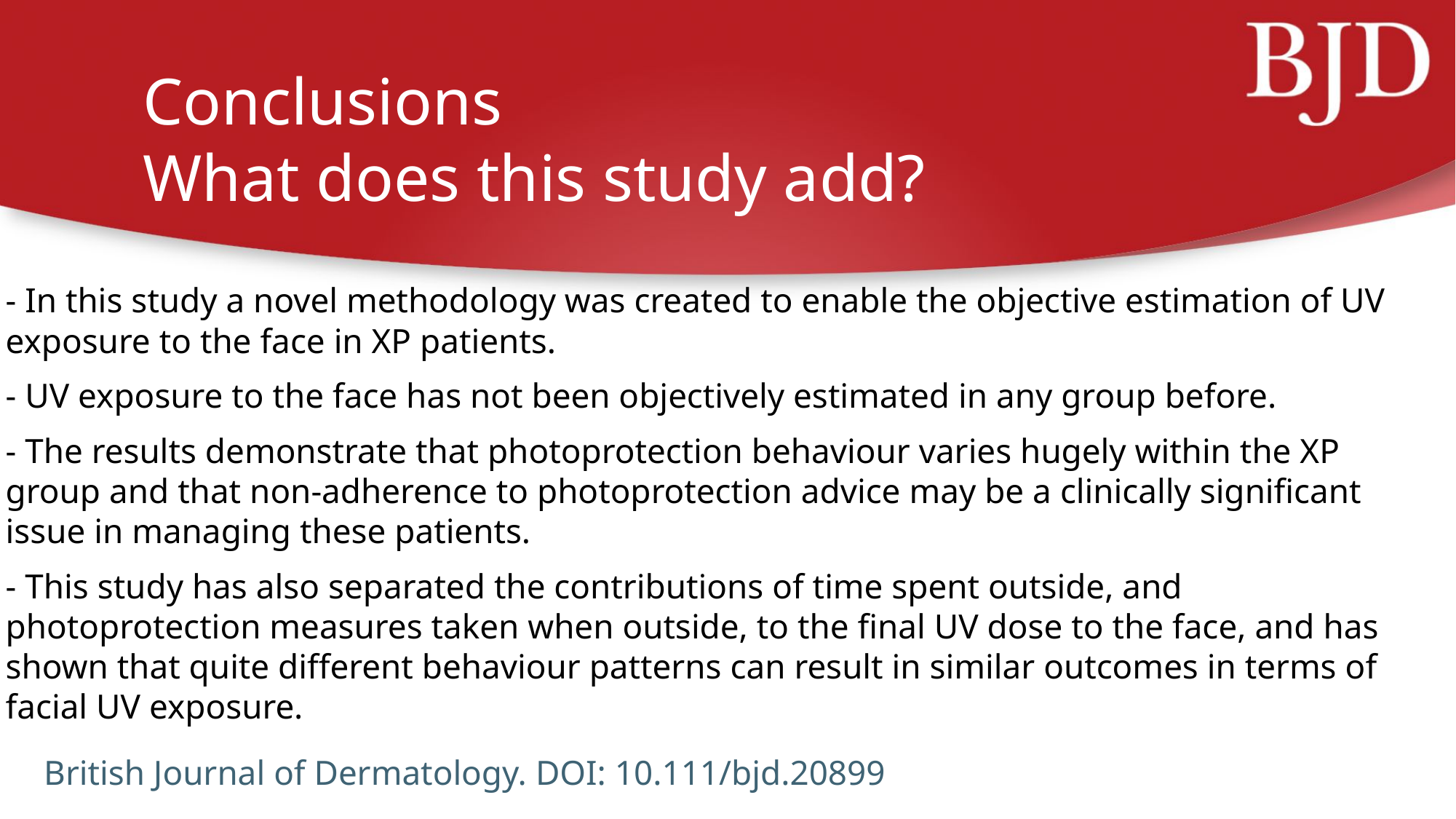

# Conclusions What does this study add?
- In this study a novel methodology was created to enable the objective estimation of UV exposure to the face in XP patients.
- UV exposure to the face has not been objectively estimated in any group before.
- The results demonstrate that photoprotection behaviour varies hugely within the XP group and that non-adherence to photoprotection advice may be a clinically significant issue in managing these patients.
- This study has also separated the contributions of time spent outside, and photoprotection measures taken when outside, to the final UV dose to the face, and has shown that quite different behaviour patterns can result in similar outcomes in terms of facial UV exposure.
British Journal of Dermatology. DOI: 10.111/bjd.20899

## Slide 14
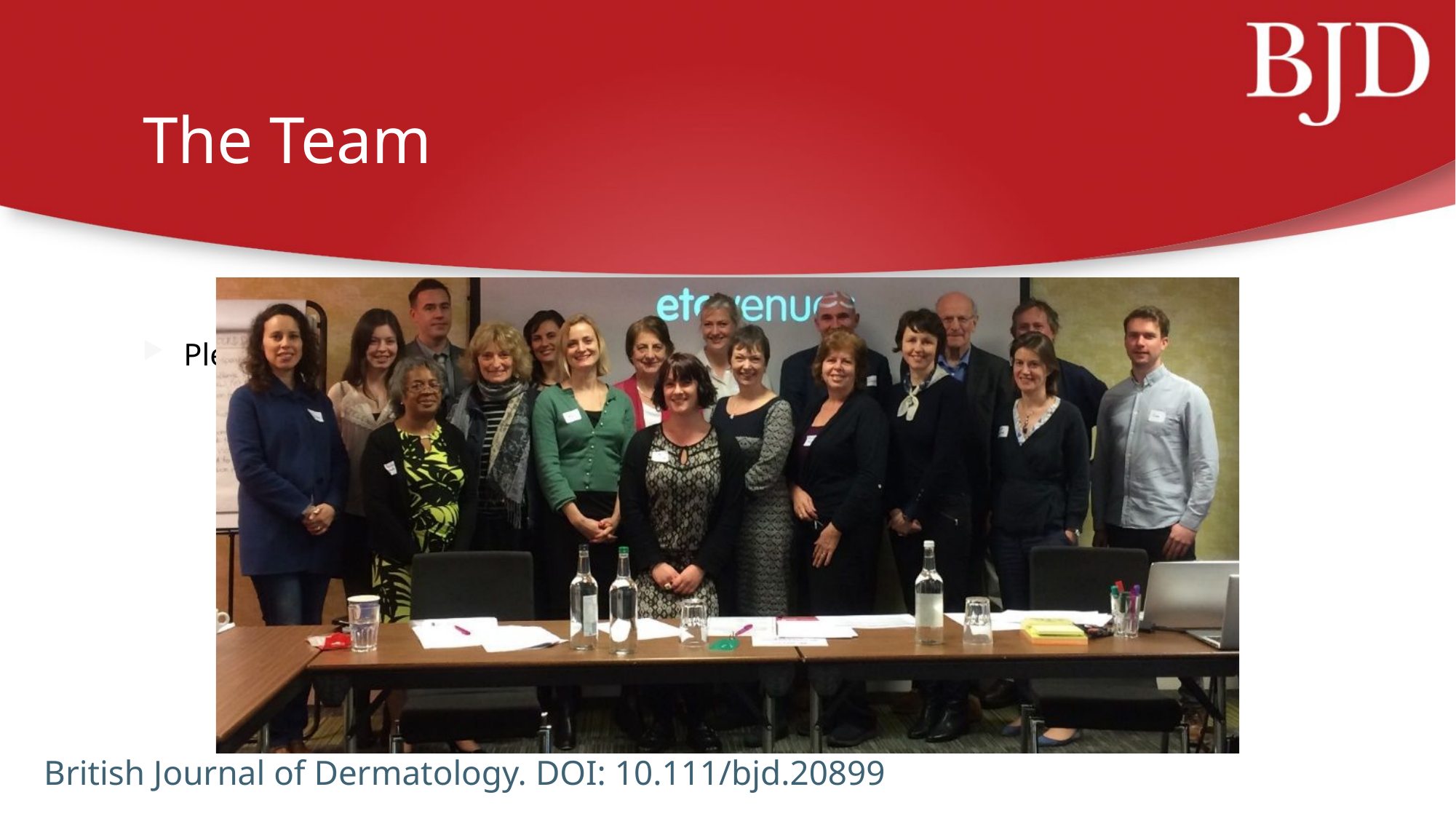

# The Team
Please add a photograph / photographs of the research team
British Journal of Dermatology. DOI: 10.111/bjd.20899

## Slide 15
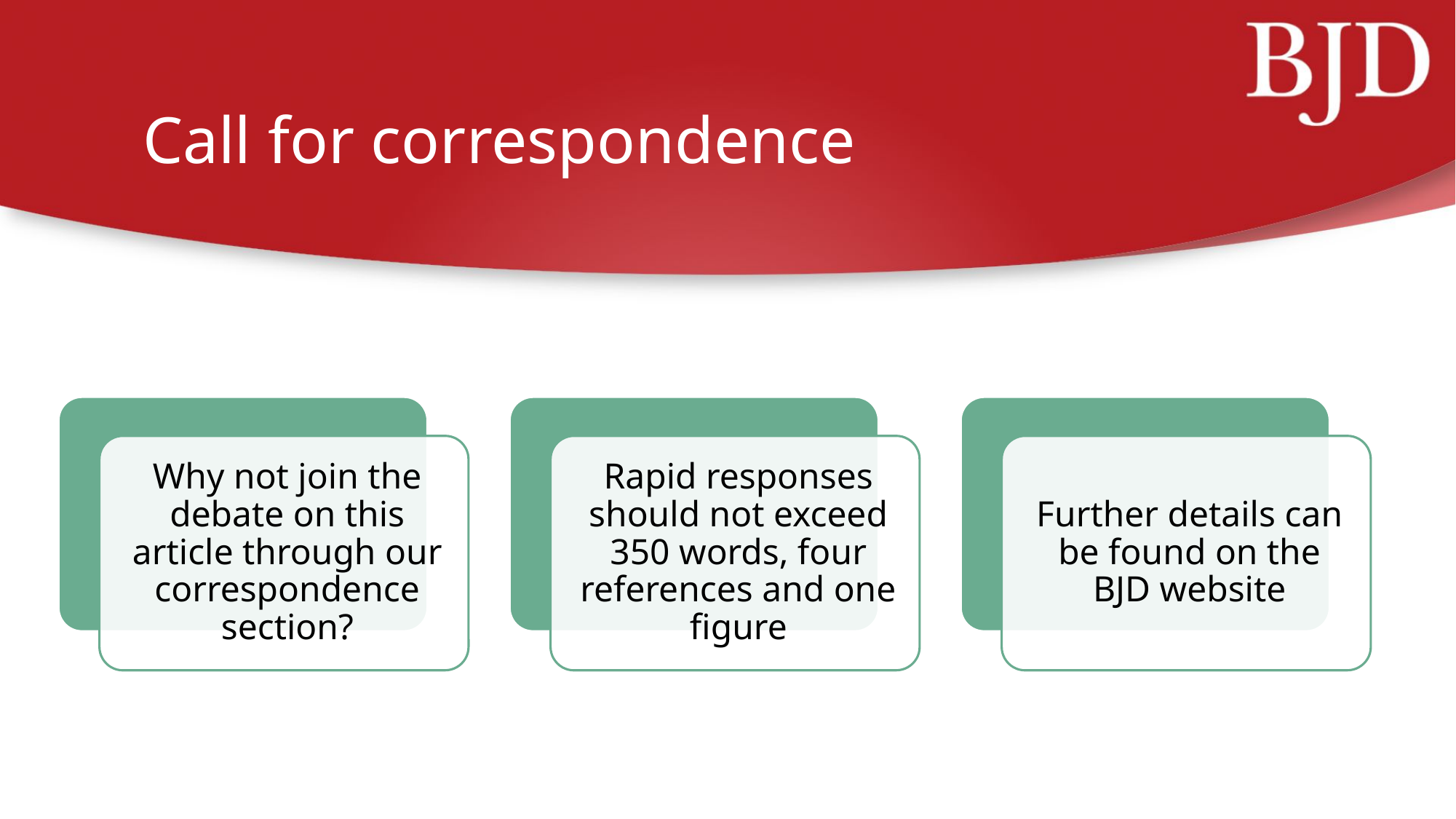

# Call for correspondence
